# A new gut pathogenic bacteria and its metabolites promote colorectal cancer development and act as non-invasive early diagnostic biomarkers

**DOI:** 10.1080/19490976.2025.2555446

**Published:** 2025-09-05

**Authors:** Rui Zhang, Mingxiao Li, Hui Tan, Jianyao Liu, Lu Wang, Wenling Dai, Zhimin Fan, Jihua Liu

**Affiliations:** a Jiangsu Key Laboratory of TCM Evaluation and Translational Research, School of Traditional Chinese Pharmacy, China Pharmaceutical University, Nanjing, Jiangsu, China; b Jiangsu Clinical Innovation Center for Anorectal Diseases of T.C.M, Nanjing Hospital of Chinese Medicine, Nanjing, China; c State Key Laboratory of Natural Medicines, School of Traditional Chinese Pharmacy, China Pharmaceutical University, Nanjing, Jiangsu, China

**Keywords:** Colorectal cancer, metabolite, diagnostic marker, intestinal bacteria, inflammatory bowel disease, DNA damage

## Abstract

Gut microbiota dysbiosis is strongly linked to colorectal cancer (CRC), but reliable early diagnostic markers remain elusive. This study investigates the role of a novel *Shigella flexneri* strain in CRC pathogenesis. Metabolomic analysis of CRC patient feces identified elevated agmatine levels. A unique agmatine-producing strain (*S. flexneri* C.11) was isolated and validated in cell models and pseudo-sterile mice. Affinity fishing combined with HPLC-QTOF-MS characterized bacterial metabolites, while RNA sequencing elucidated mechanistic pathways. Repeated *S. flexneri* C.11 exposure-induced DNA damage and inflammatory-to-neoplastic transformation. Three genotoxic cyclodipeptides (CDP1–3) were identified, driving malignant transformation and accelerating colitis-associated tumorigenesis. Mechanistically, *S. flexneri* C.11 upregulated ERBB3, activating the PI3K-AKT pathway. Clinically, combined detection of *S. flexneri* C.11 and its metabolites differentiated CRC patients from healthy controls (AUC = 0.887), suggesting its potential as noninvasive diagnostic biomarkers for CRC. We identify *S. flexneri* C.11 as a pro-carcinogenic pathogen and propose ERBB3/PI3K – AKT signaling as a therapeutic target.

## GRAPHICAL ABSTRACT

**Figure:**
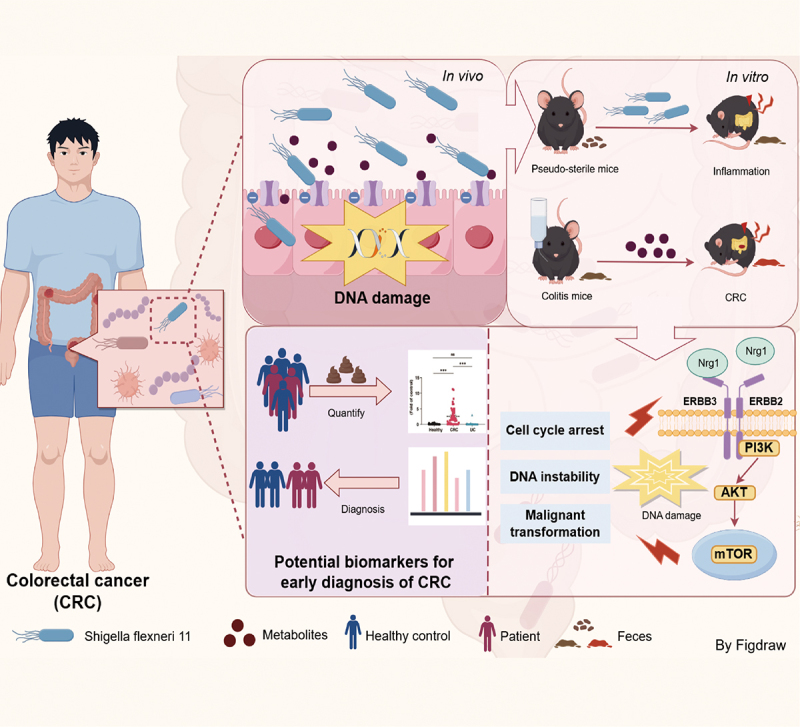

## Introduction

Colorectal cancer (CRC) ranks third among the most prevalent malignancies and is the second major cause of cancer-related fatalities,^1^ arises from complex interactions between genetic susceptibility and environmental triggers. Inflammatory bowel disease (IBD), encompassing ulcerative colitis (UC) and Crohn’s disease, plays a significant role in elevating the risk of developing CRC. Those who have IBD for more than 10 years have a 10% chance of developing cancer, and more than 20% of patients with IBD develop CRC within 30 years of diagnosis.^2,3^ However, the early symptoms of CRC are not definitive, and there are no clear markers of disease progression during the development of the inflammation-adenoma-carcinoma process.^4^ Despite advances in CRC screening, early detection remains challenging. Colonoscopy, the gold standard diagnostic tool, is invasive, costly, and exhibits limited sensitivity (20–50%) for early-stage tumors.^5^ Current noninvasive alternatives like fecal occult blood tests are hindered by low specificity and an inability to detect precancerous lesions.^6^ Therefore, the identification of sensitive and specific early markers is critical, for detecting CRC and precancerous conditions at an early stage.

Gut microbiota is one of the environmental risk factors,^7,8^ influencing the initiation, development, and metastasis of CRC.^9^ Dysbacteriosis in CRC patients is characterized by elevated fecal levels of virulent bacteria (e.g., *Escherichia coli*, *Shigella*, *Bacteroides*, and *Clostridium*) compared to healthy controls.^10^ While certain species like *Fusobacterium nucleatum* and toxigenic *Bacteroides fragilis* have established mechanistic links to CRC carcinogenesis,^11–14^ the role of *Shigella flexneri* (*S. flexneri*) – despite its significant enrichment in CRC patient tissues,^15^ remains poorly defined beyond acute inflammatory responses.^16^ Systematic investigation is still lacking regarding whether *S. flexneri* is involved in CRC initiation and progression, and through which molecular mechanisms it contributes to these processes.

To this end, this study applied untargeted metabolomics to fecal samples from CRC patients to identify differential metabolites, which enabled the isolation and characterization of the novel pro-carcinogenic *S. flexneri* strain (designated *S. flexneri* C.11). We further elucidated the molecular mechanisms by which the strain-induced DNA damage and promoted the transition from inflammation to tumorigenesis. Additionally, we evaluated the clinical potential of *S. flexneri* C.11 and its associated metabolites as noninvasive early diagnostic biomarkers, thereby providing novel perspectives for early CRC screening and therapeutic target development.

## Materials and methods

### Human samples

The diagnoses of potential patients to enroll in this study were confirmed by clinicians, based on the medical histories, physical examinations, colonoscopies, and histopathology results of the patients. Patients were included if they were 18–65 years old, diagnosed with UC or CRC, and agreed to participate in the study. Healthy controls (HCs) were age- and sex-matched with patients with UC and CRC. Individuals with other conditions impacting their metabolic profiles or physiological measurements were not included.

Serum samples were collected from 49 patients with CRC, 45 patients with UC, and 46 HCs. Fecal samples were collected from 20 patients with CRC, 22 patients with UC, and 20 HCs. Clinical CRC tissues and adjacent normal tissues (ANTs) were collected from 10 patients with CRC. Participants were asked to complete a questionnaire and sign an informed consent form. The clinical and demographic details of the study group are outlined in Supplemental Table S1. The serum and fecal samples were kept at −80°C until further analysis. Fresh tissues were fixed in 4% PFA at room temperature for 16–32 h. After embedding, sections were cut at 10–20 μm thickness for fluorescence in situ hybridization (FISH) and immunofluorescence analysis. Detailed protocols are provided in the Methods section of the Supplementary Materials. Direct consent was obtained from patient. This study was conducted in accordance with The Code of Ethics of the World Medical Association (Declaration of Helsinki). Approval for the study was obtained from the Ethics Committee of Nanjing Hospital of Chinese Medicine (approval number: KY2022306).

### Metabolomic analysis

Metabolite separation was performed using an Agilent Technologies 6530 Accurate Mass HPLC-QTOF-MS system (Santa Clara, CA, USA). Serum and fecal analyses were performed using a Zorbax extend C18 column (150 × 4.6 mm i.d., 5 µm), with a flow rate of 1.0 mL/min. The mobile phase consisted of solvent A (0.1% formic acid in water) and solvent B (acetonitrile). The chromatographic gradients were as follows: 0–50 min, 5% to 95% B; 50–60 min, 95% B; 60–70 min, 95% to 5% B. A continuous introduction of a calibrating solution was performed, containing reference masses of m/z 121.0509 (protonated purine) and m/z 922.0098 (protonated hexakis [1 H,1 H,3 H – tetrafluoropropoxy]) in positive ion mode. The mass spectrometry parameters were set as follows: fragmental voltage, 120 V; nebulizer gas, 35 psig; capillary voltage, 4 kV; drying gas flow rate, 9 L/min; and temperature, 325°C. Data were collected in the centroid and profile modes, with a mass range of 50–1,500 m/z.

Fecal sample processing: Samples were mixed with 1 mL of pre – cooled deionized water for 5 min, centrifuged at 12,000 rpm for 10 min, and the supernatant was suctioned into another Eppendorf tube. The supernatant (300 μL) was added into 10 μL of internal standard solution (final concentration, 1 μg/mL), after which 890 μL of methanol was added to precipitate the protein. After being vortexed for 5 min, the supernatant was centrifuged at 12,000 rpm for 10 min for a mass spectrometry analysis. After the absorbance had been measured, 20 μL of each sample was obtained as the quality control sample.

Serum sample processing: Whole blood (2.4 mL) was collected, placed vertically at room temperature (24°C ± 1°C) for 60 min to form clots, and centrifuged at 1,300 *g* for 10 min. Serum fractions were collected, sub-packed (50 μL per tube), and stored in a −80°C refrigerator. Next, 50 μL of each serum sample was added to 10 μL of internal standard working fluid (final concentration, 1 μg/mL), after which 140 μL of methanol was added to precipitate the protein. Following 5 min of vortexing, the samples were centrifuged at 12,000 rpm for 10 min. The resulting supernatant was then passed through a 0.22 μm filter membrane into liquid vials, ready for mass spectrometry analysis. After absorbing 20 μL of each sample, the quality control sample (QC) was obtained by full mixing.

### Isolation of agmatine-produced strains from stools and genome sequencing

Ten frozen bacterial solutions from patients with CRC were mixed with an anaerobic culture *in vitro*. Selective cultural media for *Enterococcus*, *Enterobacter*, *Streptococcus*, *Bifidobacterium*, *Clostridium*, and *Lactobacillus* were prepared. After anaerobic culture for 24 h, single colonies from each selective medium were placed into 50 mL flasks containing 20 mL of anaerobic medium and cultured for another 24 h. After centrifugation, 300 μL of supernatant was collected and mixed with 900 μL of methanol. The mixture was vortexed for 5 min to precipitate the protein, followed by recentrifugation. The resulting sample was filtered through a 0.22 μm organic filter membrane.

### Single colony selective culture

Cells from the selective medium for agmatine production were isolated and purified using the single colony method. After different colonies on the selective plate had been re-cultured and expanded, the peptone was diluted again, and the operation was repeated. After the plate had grown a single colony, different single colonies from the selective plate were selected, inoculated in 5 mL tubes, and shaken at 37°C overnight. After the bacteria had reached the logarithmic growth period, the plate was re-diluted and coated, purified once, and a single colony was selected and shaken at 37°C overnight. The fermentation broth could be made into a mass spectrum sample according to the above method. The isolated strain was stored in a −20°C refrigerator with 30% glycerol.

### Animal experiments

Azoxymethane (AOM)/dextran sodium sulfate (DSS) mice: In total, 30 C57BL/6J mice aged 6–8 weeks were randomly divided into the blank (*n* = 10) and model (*n* = 20) groups. The mice in the model group were intraperitoneally injected with AOM (10 mg/kg) and administered 2% DSS for 1 week. They were then given normal water for 2 weeks, according to a cycle of being treated every 3 weeks for three cycles. Body weights and feces were recorded twice a week to observe the state of the mice, and samples were collected at the end of the cycle.

Pseudo-sterile mice: A total of 40 C57BL/6J mice, aged 6–8 weeks, were randomly assigned to four groups: a control group (*n* = 10) and three treatment groups. The treatment group mice were administered 0.4 mL of penicillin, 0.2 g/L neomycin, 0.2 g/L metronidazole, and 0.1 g/L vancomycin every day for 14 days. After successful modeling, the animals were randomly divided into three groups: antibiotics (*n* = 10), *Escherichia coli* K12 (*E. coli* K12) (*n* = 10), and *S. flexneri* C.11 (*n* = 10). Stools were collected twice a week, and weight changes were recorded. After 14 days of antibiotic intervention, most of the intestinal flora was removed to facilitate the transplantation of control and pathogenic bacteria. The logarithmic-phase *E. coli* K12 and *S. flexneri* C.11 were rejuvenated and centrifuged at 12,000 rpm for 10 min, after which the supernatant was removed and suspended in sterilized phosphate-buffered saline (PBS). The cell density was approximately 10^9^ cfu/mL. Each mouse was fed with 0.2 mL of 2 × 10^8^ cfu per day, and the control and antibiotic groups were fed an equal amount of PBS. Feces were collected twice a week at weeks 6 and 14 after transplantation, and weight changes were recorded.

Colitis mice: In total, 60 C57BL/6J mice aged 6–8 weeks were randomly divided into six experimental groups (*n* = 10): (1) control group: normal control with daily gavage of normal saline; (2) AOM-DSS group: as mentioned before; (3) DSS group: 2% DSS with daily gavage of normal saline; and (4) cyclodipeptides (CDPs) group: 2% DSS plus daily gavage of cyclo (Pro-Leu) (CDP1) (20 mg/kg), cyclo (Phe-Pro) (CDP2) (20 mg/kg), or cyclo (Pro-Val) (CDP3) (20 mg/kg). The control group was given drinking water, whereas the other groups were given drinking water containing 2.0% DSS for 7 days in three cycles.

All animal procedures in this study were approved by the Animal Ethics Committee of China Pharmaceutical University (2024–06–32). Samples from live and dead animals were collected in strict accordance with the recommendations of the Committee on Animal Welfare.

### Statistical analysis

All statistical tests were performed using GraphPad Prism v8.0 (GraphPad Software, La Jolla, CA, USA). All values are expressed as the mean ± standard error of the mean (SEM). A two-tailed Student’s *t*-test was used for comparisons between two groups. Results with *p* < 0.05 were considered statistically significant. More detailed methods and materials can be found in the supplementary materials.

## Results

### Differential metabolite-associated microbiota in CRC

**Figure 1.**
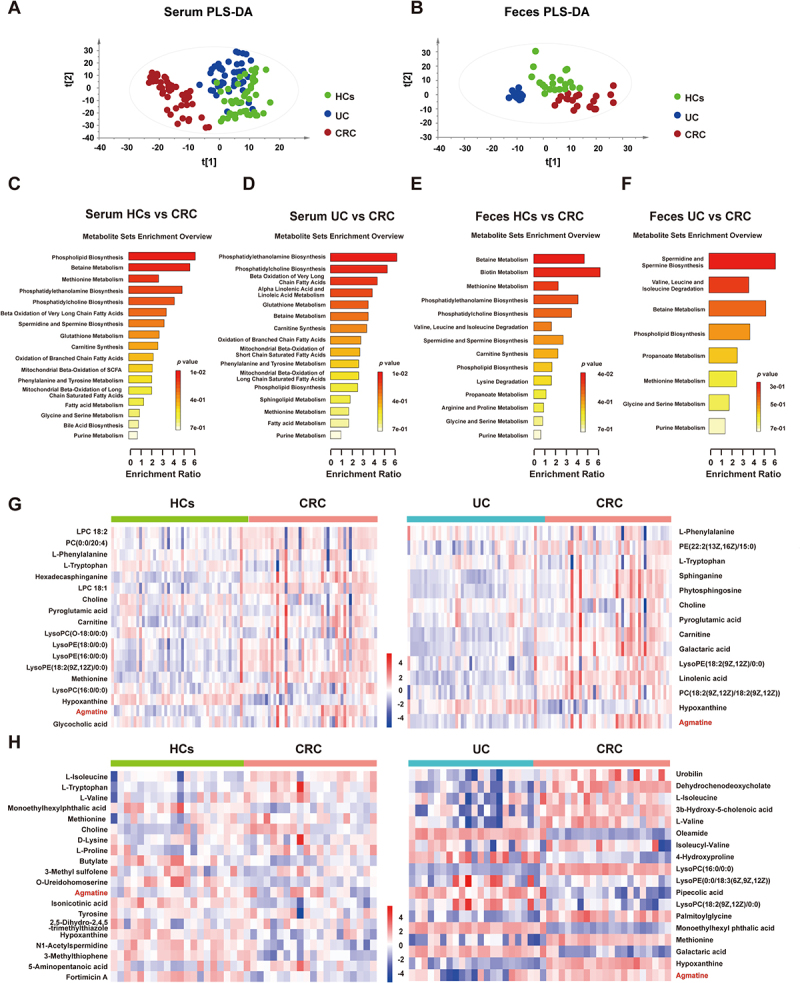
Metabolic alterations in CRC are found in serum and feces samples.

Using non-targeted metabolomics, serum samples from 140 participants (46 HCs, 45 UC patients, and 49 CRC patients) and stool samples from 62 participants (20 HCs, 22 UC patients, and 20 CRC patients) were analyzed to screen for differentially expressed metabolites. A partial least squares discriminant analysis (PLS-DA) revealed that both serum and stool samples from patients with CRC could be distinguished from those from HCs and patients with UC, suggesting the presence of metabolic disorders in patients with CRC (Figure 1(A,B)). Seventy differential metabolites were deemed statistically significant with a *p* value < 0.05 and variable importance in projection (VIP) > 1, analyzed using MetaboAnalyst online software for multivariate assessments (Table S2). Using the Kyoto Encyclopedia of Genes and Genomes (KEGG) database, key metabolic pathways altered in patients with CRC were identified. The pathways identified encompassed methionine, biotin, betaine metabolism, glycine and serine metabolism, and valine, leucine, L-isoleucine degradation (Figure 1(C–F)). A heatmap was employed for hierarchical clustering to illustrate the concentration variations in potential biomarkers among CRC patients, UC patients, and HCs (Figure 1(G–H)). Serum and fecal samples from patients with CRC contained higher levels of agmatine than those of HCs and patients with UC (Figure S1(A,C)). Furthermore, receiver operating characteristic (ROC) curve analysis revealed that the area under the curve (AUC) values for agmatine exceeded 0.7 in both sample types (Figures S1(B,D)), suggesting its potential diagnostic value for CRC. Agmatine, a neuroprotective arginine metabolite with minimal endogenous production, is associated with the intestinal flora.^17^ To confirm the role of the gut microbiota in agmatine metabolism, we used an *in vitro* gut simulator inoculated with feces from patients with CRC. After 24 h of culture, the agmatine content in the media significantly increased, confirming that the gut microbiota could be metabolized to produce agmatine (Figure S1(E)). We also conducted *in vivo* experiments to confirm the involvement of gut microbiota in the generation of agmatine. Normal mice treated with a combination of antibiotics to clear their intestinal flora exhibited significantly decreased agmatine levels (Figure S1(F)). Similarly, in the AOM – DSS-induced colitis – associated cancer model (detailed in Figure S2), the agmatine content in the feces gradually increased as the disease progressed, and the difference was significant (Figure S1I). In addition, the DNA damage potential of agmatine was assessed. The expression of γH2AX (a DNA damage marker) was significantly upregulated only at 1 μg/mL agmatine (Figure S1(G)). Clinical fecal study revealed physiological agmatine levels in humans were 0.13–275 ng/mL (Figure S1(H)), far below the DNA damage-inducing threshold, suggesting no direct genotoxicity under normal conditions.

(A) PLS-DA score plots for serum. HCs (*n* = 46) are shown in green; UC patients (*n* = 45) are shown in blue and CRC patients (*n* = 49) are shown in red. (B) PLS-DA score plots for feces. HCs (*n* = 20) are shown in green; UC patients (*n* = 22) are shown in blue and CRC patients (*n* = 20) are shown in red. (C) Overview of pathways related to the differential endogenous metabolites in serum between HCs and CRC patients. (D) Overview of pathways related to the differential endogenous metabolites in serum between UC patients and CRC patients. (E) Overview of pathways related to the differential endogenous metabolites in feces between HCs and CRC patients. (F) Overview of pathways related to the differential endogenous metabolites in feces between UC patients and CRC patients. (G) Heatmap of the differential metabolites between the HCs, UC patients and CRC patients in serum. Red represented the metabolites in high abundance; blue represented the metabolites in low abundance. (H) Heatmap of the differential metabolites between the HCs, UC patients and CRC patients in feces. Red represented the metabolites in high abundance; blue represented the metabolites in low abundance.

### Identification of a new *Shigella flexneri* strain, *S.* *flexneri* C.11

**Figure 2.**
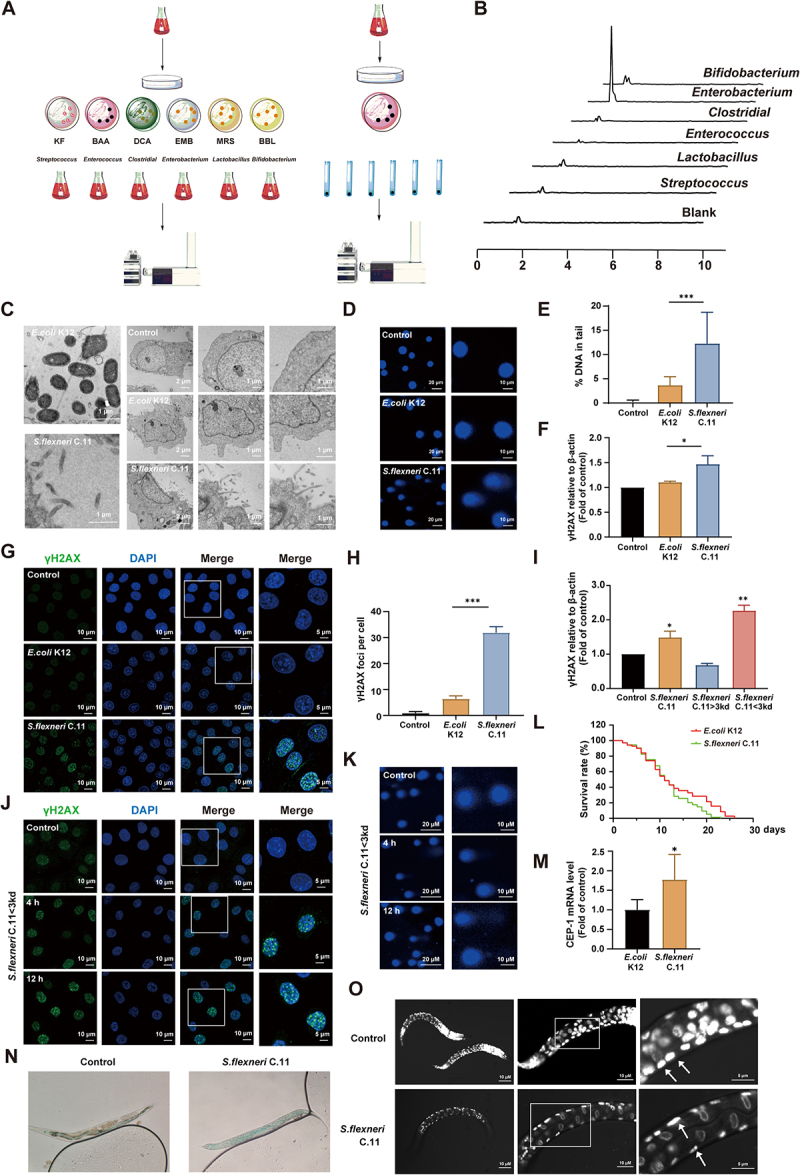
*S. flexneri* C.11 causes DNA damage *in vitro*, but not through agmatine.

The selective cultivation of the intestinal microbiota of patients with CRC was conducted to identify the agmatine-producing genera (Figure 2(A)). Quantitative analysis revealed *S. flexneri* as the predominant agmatine-producing genus (Figure 2(B)). A novel strain *S. flexneri* C.11 was isolated, purified, and characterized by mass spectrometry and whole-genome sequencing (GTDB-tk database; Figure S3). Comparative genomic analysis with pks^+^ *E. coli*, a known CRC-associated strain producing colibactin, that confirmed the absence of colibactin biosynthetic islands in *S. flexneri* C.11. Thus, the results indicating that it may be an unreported carcinogenic *S. flexneri* with serotypes of 2b (Table S3), and nomenclated as ‘*S. flexneri* C.11.’ Transmission electron microscopy revealed that *S. flexneri* C.11 was cell-invasive, with direct adhesion to and penetration of host cells (Figure 2(C)), a hallmark of pathogenic *Shigella*.^18^ To evaluate its genotoxic potential, the comet assay and immunofluorescence analyses were performed. *S. flexneri* C.11 induced pronounced DNA double-strand breaks, evidenced by elongated DNA tails (Figure 2(D,E)) and elevated γH2AX expression after 4 h co-incubation (Figure 2(F–H)). To further determine whether live bacteria or their metabolites exert DNA damage, IEC-6 cells were treated separately with the different part culture medium of *S. flexneri* C.11 based on its molecular weight. The results demonstrate that the low-molecular-weight fraction ( < 3 kd) elevated γH2AX expression levels (Figure 2(I)), with DNA damage severity escalating over time (Figure 2(J,K)). In order to validate the *in vivo* pathogenicity of *S. flexneri* C.11, *Caenorhabditis elegans* (*C. elegans*), a well-established model was used for studying host-microbiota interactions.^19^ Nematodes fed *S. flexneri* C.11 exhibited a significantly shortened lifespan compared to controls (Figure 2(L)), accompanied by severe intestinal barrier dysfunction, as demonstrated by dye leakage assays (Figure 2(N)). *S. flexneri* C.11 triggered transcriptional upregulation of *CEP-1*, the *C. elegans* homolog of the tumor suppressor *p53* (Figure 2(M)), and induced asymmetric nuclear division in intestinal cells of *C. elegans* (Figure 2(O)). In conclusion, these findings demonstrated that *S. flexneri* C.11 not only exhibited cellular invasiveness, but also produced metabolites that activated the DNA damage response, and suggesting its potential carcinogenic risk.

(A) Schematic diagram of selective culture operation. (B) Screening of strains producing agmatine. (C) Representative transmission electron microscopy images of *S. flexneri* C.11 attaching and invading into IEC-6 cells. *E. coli* K12 was used as the negative control. (D) Comet assay in IEC–6 cells treated with *E. coli* K12 and *S. flexneri* C.11. (E) Histograms showing %DNA in tail. (F) Western blot of γH2AX expression in IEC-6 cells treated with *E. coli* K12 and *S. flexneri* C.11. (G) Representative images of γH2AX immunofluorescence staining in IEC-6 cells treated with *E. coli* K12 and *S. flexneri* C.11. (H) Immunofluorescence histograms showing γH2AX foci per cell. (I) Western blot of γH2AX expression in IEC-6 cells treated with different part culture medium of *S. flexneri* C.11 for 4 h. (J) Representative images of γH2AX immunofluorescence staining in IEC-6 cells treated with *S. flexneri* C.11 < 3kd for 4 h and 12 h. (K) Comet assay in IEC-6 cells treated with *S. flexneri* C.11 < 3kd for 4 h and 12 h. (L) The survival curves in *C. elegans* treated with *S. flexneri* C.11. *E. coli* OP50 served as the negative control as it represents the standard food for *C. elegans*. (M) The mRNA levels of *CEP-1* detected by RT-qPCR in *C. elegans* treated with *S. flexneri* C.11. (N) Representative images of *S. flexneri* C.11 breaks down the intestinal barrier of *C. elegans* and cause dye leakage. (O) Fluorescent images of *C. elegans* fed on *S. flexneri* C.11 from the time of hatching, white arrowheads denote asymmetrical karyokinesis. *p < 0.05, **p < 0.01, ***p < 0.001; NS, not significant.

### *S.* *flexneri* C.11 induces intestinal inflammation and accelerates its transformation to cancer in mice

**Figure 3.**
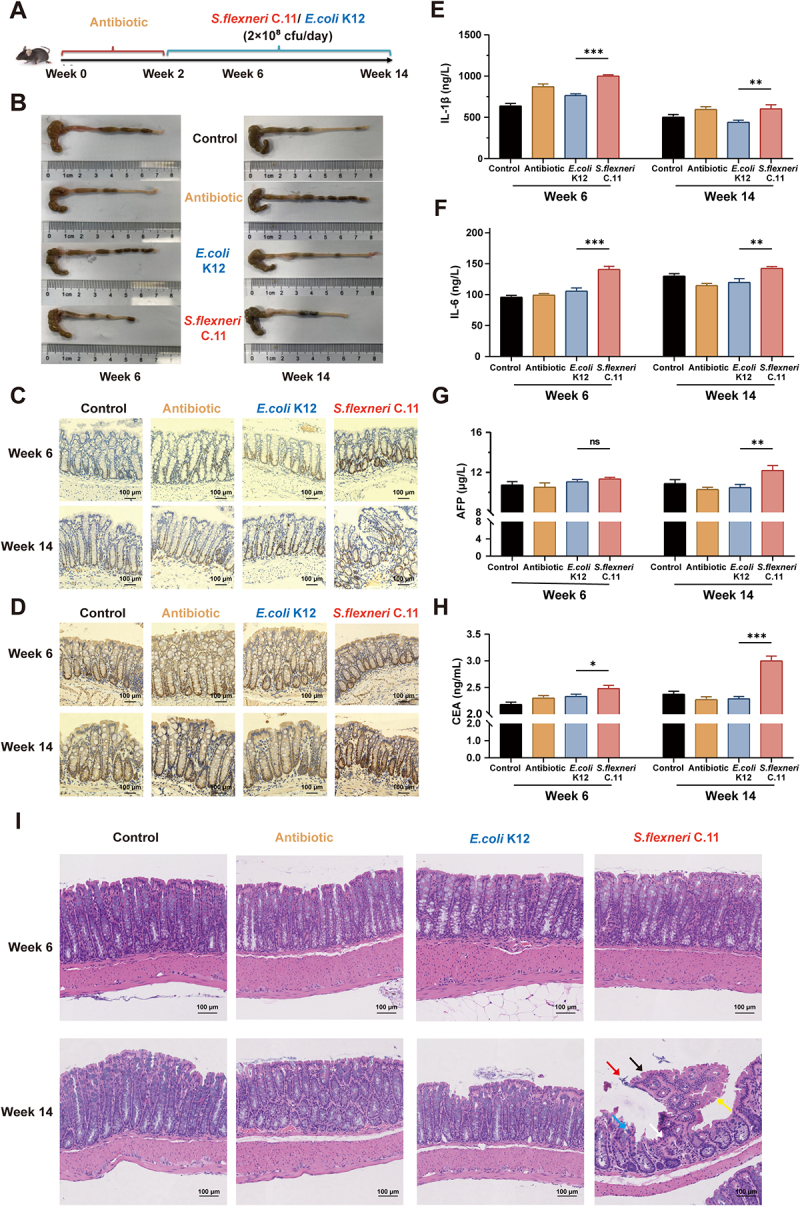
*S. flexneri* C.11 induces intestinal inflammation and accelerates its transformation to cancer in mice.

To determine the oncogenic potential of *S. flexneri* C.11, avirulent *E. coli* K12 was used as a control. The mice were treated with a composite antibiotic for 2 weeks to clear the most intestinal flora within the gut. 16S rDNA high-throughput sequencing revealed significant differences in α-diversity and principal coordinate analysis (PCoA) between untreated and antibiotic-treated groups (Figure S4(A – E)), confirming successful establishment of a pseudo-sterile mice model. Subsequently, *S. flexneri* C.11 and the control strain *E. coli* K12 were transplanted (Figure 3(A)). While no significant differences in body weight were observed at 6- or 14-weeks post-colonization (Figure S4F), the colonization of *S. flexneri* C.11 exhibited significantly shorter colons compared to controls at both time points (Figure 3(B), Figure S4(G)), suggesting a potential pro-inflammatory role for *S. flexneri* C.11.

(A) Schematic diagram showing the experimental design. A cohort of C57BL/6J mice (*n* = 6 per group) were treated with antibiotic for 2 weeks and immediately gavaged with a single dose (2 × 10^8^ cfu every day) of *S. flexneri* C.11 or *E. coli* K12 (or PBS in control group) for 12 weeks. (B) Macroscopic morphologies of colon sections in each group. (C – D) PCNA and Ki67 immunohistochemistry from mice in each group. (E) Serum IL-1β, (F) Serum IL-6, (G) serum AFP and (H) serum CEA were measured after 6 and 14 weeks of treatment. (I) H&E staining colon sections of mice in each group. Data, mean ± SEM. *p < 0.05, **p < 0.01, ***p < 0.001; NS, not significant.

To confirm this result, the levels of pro-inflammatory cytokines IL-1β and IL-6 in mouse serum were measured by enzyme-linked immunosorbent assays.^20^ *S. flexneri* C.11 significantly upregulated serum levels of IL-1β and IL-6 after 6 weeks compared with the control (Figure 3(E,F)), suggesting a pro-inflammatory effect of *S. flexneri* C.11. We further noted an upregulation in the serum levels of carcinoembryonic antigen (CEA) and alpha-fetoprotein (AFP), which are commonly used clinical cancer markers, following *S. flexneri* C.11 treatment for 6 weeks, with a more significant difference at 14 weeks (Figure 3(H)). AFP was notably upregulated after 14 weeks (Figure 3(G)), suggesting a pro-inflammatory transformation role for *S. flexneri* C.11. After 14 weeks but not 6 weeks of *S. flexneri* C.11 transplantation, hematoxylin-eosin (H&E) staining results showed the intestinal lumen showed proliferative protrusions (black arrowheads), rough and uneven striated edges (yellow arrowheads) on the surfaces of intestinal mucosal epithelial cells, as well as dense epithelial cells with slightly irregular and short columnar arrangements (white arrow), necrotic epithelial cells (red arrow), and fibrin exudates (blue arrow) in the lumen (Figure 3(I), Figure S4(J)). At 6 weeks, Ki67 and proliferating cell nuclear antigen (PCNA) levels were significantly upregulated compared to those in the controls and remained high at 14 weeks (Figure 3(C,D), Figure S4(H – I)). *S. flexneri* C.11, therefore, promotes the development of intestinal inflammation in mice and exhibits a tendency to promote cancer progression.

### Genotoxic metabolites from *S.* *flexneri* C.11 induce cellular malignant transformation

**Figure 4.**
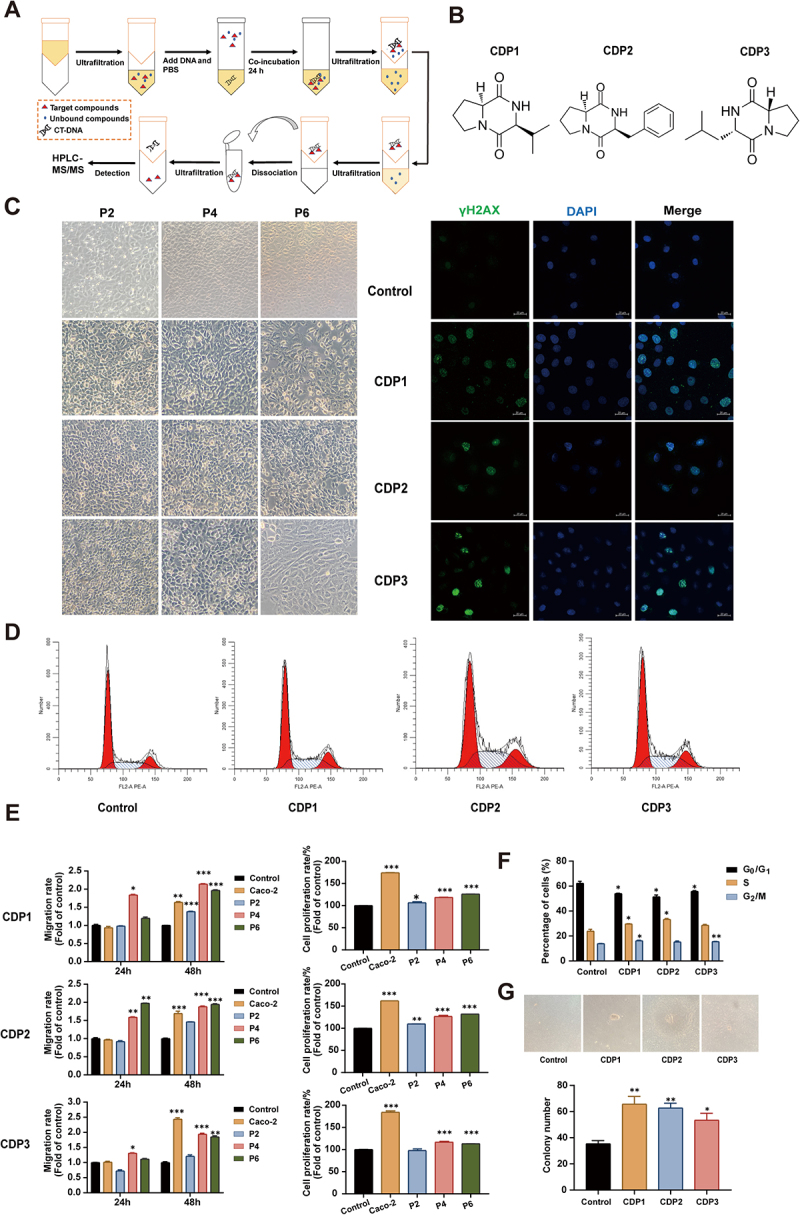
Direct effects of *S. flexneri* C.11 genotoxic metabolites *in vitro*.

As previously described, the concentration of agmatine, a metabolite derived from *S. flexneri* C.11, induces DNA damage in IEC-6 cells at concentrations exceeding physiological levels observed in humans. Further verification revealed that the fermentation broth of *S. flexneri* C.11 possessed notable DNA-damaging capabilities, indicating that *S. flexneri* C.11 might produce other unidentified DNA-damaging metabolites. Therefore, the ligand fishing combined with HPLC-QTOF-MS was performed to screen for *S. flexneri* C.11 metabolites capable of directly interacting with DNA (Figure 4(A)). Calf thymus DNA (CT-DNA) was employed as the binding substrate, representing a well-characterized model system for investigating DNA-interactive compounds.^21^ Multiple putative DNA-binding candidates, including amino acids and peptides, were identified by dissociating the DNA adducts, as shown in Supplemental Table S4. Three CDPs were regarded as potential ligands for binding to CT-DNA, using the MassBank and Global Natural Product Social Molecular Networking (GNPS) websites and standard comparisons. These CDPs, including cyclo (Pro-Leu) (CDP1), cyclo (Phe-Pro) (CDP2), and cyclo (Pro-Val) (CDP3), were considered genotoxic metabolites for follow-up studies. Their structural formulae are shown in Figure 4(B). Each CDP was tested for its ability to damage DNA and was shown to dramatically enhance γH2AX production in a dose – dependent manner (Figure S5(A–B)).

(A) Ligand-fishing strategy screening assay schedule. (B) Structural formula of genotoxic metabolites. (C) Representative images of cell morphology (×100) and γH2AX immunofluorescence. (D) Cell cycle distribution after CDPs exposure at passage 6. (E) Cell proliferation rate and migration rate. (F) Statistical histogram of cell cycle distribution. The percentages of cell numbers in each phase were calculated from individual histograms in at least three independent experiments (*n* = 3). (G) Representative images and statistical histograms of soft agar colony formation experiments (×100). Data, mean ± SEM. *p < 0.05, **p < 0.01, and ***p < 0.001 indicate significant differences compared to control.

To further investigate direct effects of *S. flexneri* C.11 metabolites on human colorectal inflammatory cancer transformation and tumor development, we treated IEC-6 cells with 100 μM CDPs for six consecutive passage^22^. Owing to continuous administration and passage, the cell growth rate of the CDP group accelerated, leading to significant morphological changes accompanied by a loss of contact inhibition. In particular, the cell morphology changed from a normal round and spherical morphology to a spindle – like mesenchymal morphology, and the cell arrangement became chaotic and overlapped (Figure 4(C)). Moreover, continuous CDP exposure increased intracellular γH2AX activation and production of DNA double-strand breaks (Figure S5C). The immunofluorescent detection of γH2AX in the nucleus revealed an increase in the number of γH2AX foci in the nuclei of the cells in the CDP-treated group (Figure 4(C)). Flow cytometry revealed that CDPs exposure led to a marked increase in S phase cells and a notable decrease in G_2_/M phase cells, accelerating the cell cycle from G_0_/G_1_ to S and G_2_/M and causing abnormal cell proliferation (Figure 4(D,F)). Additionally, exposure to CDPs significantly enhanced the proliferation and migration abilities of the cells (Figure 4(E)). CDP1 and CDP2 were capable of forming colonies on soft agar, whereas normal IEC-6 cells and CDP3-infected cells were unable to form cell clones (Figure 4(G)), indicating that cells transformed by CDP1 and CDP2 administration exhibited non-anchorage-dependent growth and acquired some of the biological properties of malignant cells.

### Genotoxic metabolite CDPs facilitate the development of internal tumors in colitis mice models

**Figure 5.**
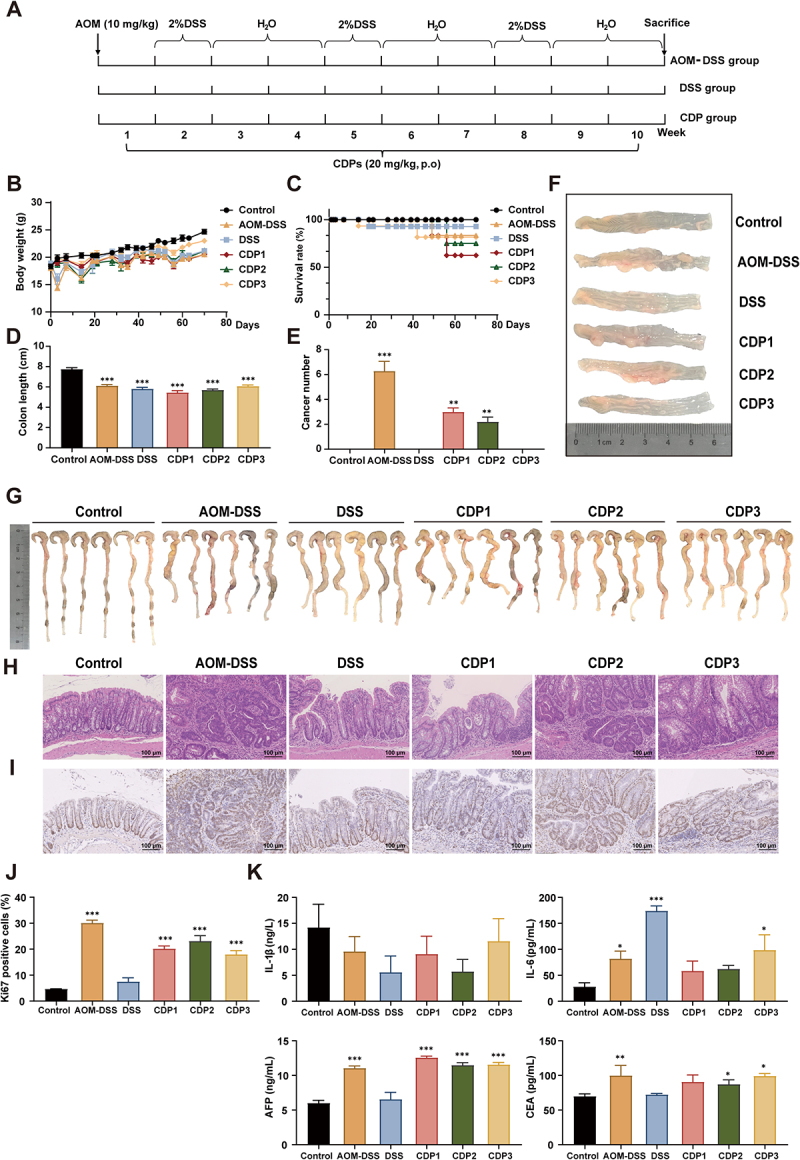
*S. flexneri* C.11 genotoxic metabolite CDPs facilitate the development of internal tumor in colitis mice.

To directly assess the role of CDPs in colitis-associated CRC, we orally inoculated mice with CDPs or saline during DSS-induced colitis (Figure 5(A)). The AOM-DSS, DSS, CDP1, and CDP2 groups experienced significant decreases in body weight compared with the control group (Figure 5(B)). Notably, the CDP1 group lost more weight throughout the second and third rounds of DSS treatment than did the other CDP groups, and its survival rates were significantly reduced (Figure 5(C)). After 10 weeks of DSS treatment, the colons of mice in all groups were significantly shorter (Figure 5(D,G)). Additionally, varying numbers of tumor masses were observed in the tissues of the AOM-DSS, CDP1, and CDP2 groups (Figure 5(E,F)). Hematoxylin and eosin (H&E) staining revealed that the AOM-DSS mice had increased leukocyte infiltration, mucosal ulceration, crypt disruption, and focal high-grade intraepithelial neoplasia with malignant transformation (mucosal carcinoma) in the colorectum (Figure 5(H)). Moreover, the DSS mice treated with CDPs developed adenomatous lesions with low-grade dysplasia. Ki67 staining showed that the percentage of positive areas was considerably higher in the AOM-DSS and CDP groups (Figure 5(I,J)). IL-1β and IL-6 serum levels were significantly higher in the model and CDP groups, while CEA and AFP levels exhibited similar trends (Figure 5(K)), indicating that CDPs facilitate the development of internal tumors in colitis mice models.

(A) The establishment scheme of the colitis model. (B) Body weight changes of mice (*n* = 10 per group). (C) Survival curve of mice. (D) Colon length (cm). (E) Tumor number. (F) Tumor load of mice. (G) Representative colon length images. (H) H&E staining (I, J) Ki67 immunohistochemical staining and quantification in mice. (K) Serum IL-1β, IL-6, AFP and CEA were measured by Elisa. Data, mean ± SEM. *p < 0.05, **p < 0.01, and ***p < 0.001 indicate significant differences compared to control.

### *S.* *flexneri* C.11-induced tumorigenesis via activation of the ERBB3/NRG1/PI3K– AKT signaling pathway

**Figure 6.**
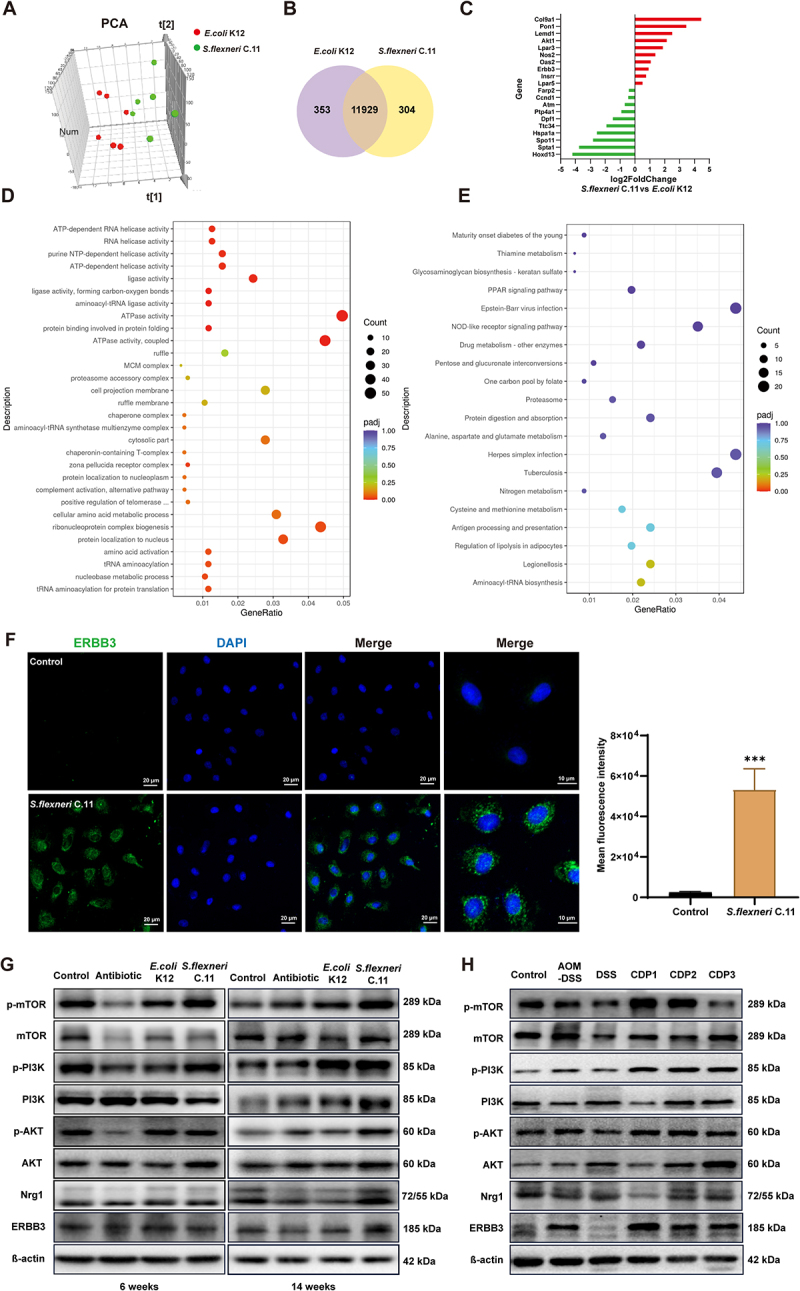
*S. flexneri* C.11-induced tumorigenesis links to host gene expression alterations.

To better understand the molecular mechanisms by which *S. flexneri* C.11 and CDPs promote the progression of colitis to CRC, transcriptome sequencing was performed on *S. flexneri* C.11 and *E. coli* K12 groups at 14 weeks. A principal component analysis (PCA) (Figure 6(A)) revealed a significant difference in the transcriptomes of the two groups, dividing them into two distinct parts. Among them, 674 genes were upregulated and 666 genes were downregulated (Figure 6(B)). Figure 6(C) illustrates differentially expressed genes between *S. flexneri* C.11 and control groups, showing significant upregulation of ERBB3 and AKT core pathway targets compared to normal strains. Further analysis of the differentially expressed genes using the gene ontology (GO) and KEGG databases (Figure 6(D,E)) revealed significant differences in the pathways associated with tRNA aminoacylation, base metabolism, ATPase activation, and aminoacyl ligase activation.

RNA from distal colon tissues of mice infected with *S. flexneri* C.11 (*n* = 6) and controls (*n* = 6) were subject to RNA-seq. (A) PCA between *S. flexneri* C.11 group and control group. (B) Venn plot between *S. flexneri C.11* group and control group. (C) Transcriptomic differential gene expression analysis. (D) GO enrichment analysis scatter plot. (E) KEGG enrichment analysis scatter plot (F) Representative images of ERBB3 immunofluorescence staining in IEC-6 cells treated with *S. flexneri* C.11. *E. coli* K12 was used as the negative control. (G) Western blotting results of ERBB3/Nrg1 expression and PI3K-AKT-mTOR pathway in intestinal tissue of pseudo-sterile mice at 6 and 14 weeks, respectively. (H) Western blotting results of ERBB3/Nrg1 expression and PI3K-AKT-mTOR pathway in intestinal tissue of colitis mice treated with CDPs. *p < 0.05, **p < 0.01, and ***p < 0.001 indicate significant differences compared to control.

ERBB3, a member of the epidermal growth factor receptor (EGFR) family, is a tyrosine kinase receptor that has been implicated as a disease driver in a number of solid tumors.^23–25^ The neuregulin 1 (Nrg1) protein acts as a direct ligand for ERBB3 and ERBB4 tyrosine kinase receptors.^26^ Once Nrg1 binds to ERBB3, ERBB2 can phosphorylate ERBB3 C-terminal tyrosine residues by transphosphorylation. The regulatory subunit of PI3K is recruited to multiple phosphoserine residues in the ERBB3 cytoplasmic domain, resulting in the strong activation of the PI3K/AKT survival pathway^27–29^ The immunofluorescence results showed that after treatment with *S. flexneri* C.11 there was a significant upregulation in the expression of ERBB3 (Figure 6(F)). Western blotting showed that ERBB3, Nrg1, p-PI3K, and p-mTOR protein expression was upregulated at 6 weeks, and a significant upregulation of p-PI3K, Ngr1, p-AKT, and p-mTOR protein expression was observed after 14 weeks (Figure 6(G)). Additionally, CDPs could also directly activate ERBB3 and the expression of key downstream proteins in the PI3K/AKT pathway (Figure 6(H)). These results suggest that *S. flexneri* C.11 exerts its biological activity by activating ERBB3 and recruiting Nrg1 receptors to induce PI3K/AKT/mTOR phosphorylation and activate downstream intracellular signaling pathways.

### Clinical correlative validation of the *S.* *flexneri* C.11-ERBB3-DNA damage pathway in CRC

**Figure 7.**
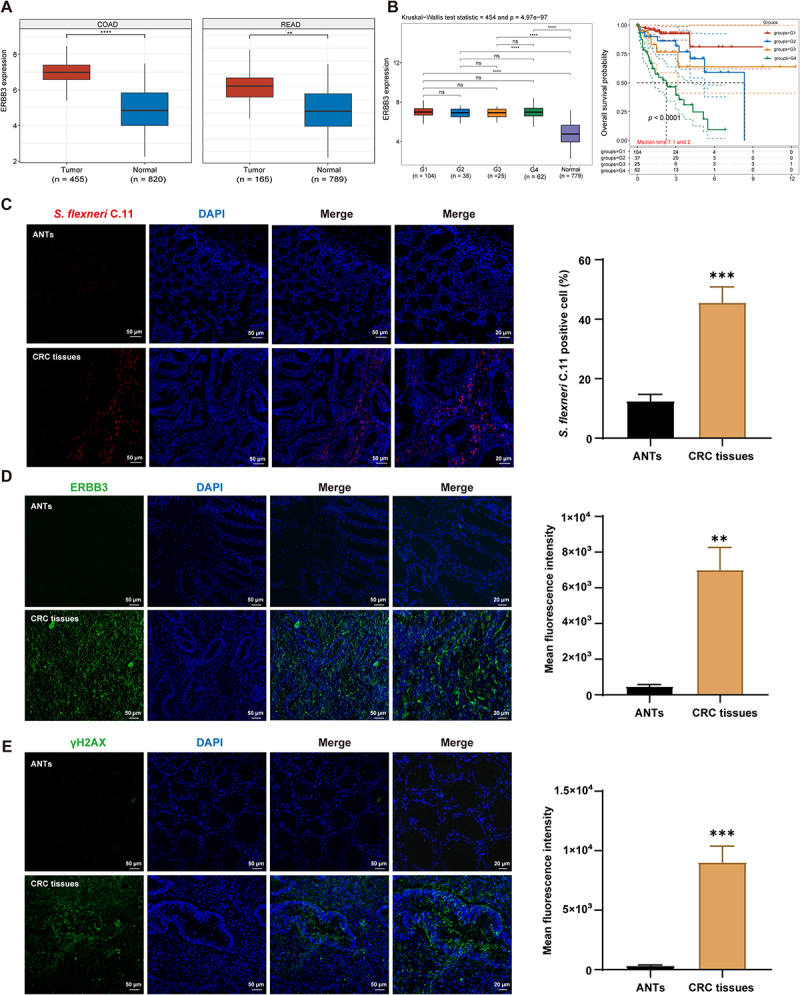
Clinical correlative validation of the *S. flexneri* C.11-ERBB3-DNA damage pathway in CRC.

To systematically elucidate the clinical significance of the *S. flexneri* C.11-ERBB3-DNA damage pathway in CRC progression, we conducted a series of validation studies. First, an integrated bioinformatics analysis of The Cancer Genome Atlas (TCGA) and Genotype-Tissue Expression (GTEx) databases revealed that ERBB3 was significantly upregulated in both colon adenocarcinoma (COAD) and rectal adenocarcinoma (READ) tissues (*p* < 0.001, *p* < 0.0001, Figure 7(A)), and its high expression was significantly associated with shorter overall survival in CRC patients (*p* < 0.0001, Figure 7(B)). Notably, ERBB3 expression showed no significant differences across pathological stages (Figure 7(B)), which closely mirrored the abundance dynamics of *S. flexneri* C.11 during CRC progression (Figure S6(A)). These results suggest that *S. flexneri* C.11 may drive the inflammation-to-cancer transition through the activation of the ERBB3 pathway in early carcinogenesis, while during tumor progression, the sustained activity of this pathway may be maintained by the interplay between the bacterial and host microenvironment.

(A) Bioinformatic analysis of ERBB3 expression in TCGA and GTEx databases. (B) Stage-dependent ERBB3 expression and survival correlation in CRC. (C) Representative fish images and quantification of *S. flexneri* C.11 colonization in clinical tissue samples. (D – E) Immunofluorescence analysis of ERBB3 and γH2AX expression in clinical tissues with statistical quantification (*n* = 8). ANTs: Adjacent normal tissues. *p < 0.05, **p < 0.01, and ***p < 0.001 indicate significant differences compared to control.

For further validation, we performed FISH to assess *S. flexneri* C.11 colonization in clinical CRC tissues and ANTs. The results demonstrated a significantly higher abundance *S. flexneri* C.11 in CRC tumor tissues than in ANTs (*p* < 0.001, Figure 7(C)). Further immunofluorescence analysis confirmed that both ERBB3 (*p* < 0.01) and the γH2AX (*p* < 0.001) were significantly upregulated in CRC tissues (Figure 7(D,E)). Importantly, Spearman correlation analysis revealed strong positive associations between *S. flexneri* C.11 colonization density and ERBB3 expression (*r* = 0.61, *p* < 0.05) as well as γH2AX levels (*r* = 0.56, *p* < 0.05) (Figure S6B – C). A significant correlation was also observed between ERBB3 and γH2AX in tumor tissues (*r* = 0.60, *p* < 0.05, Figure S6(D)), whereas no statistically significant correlations were detected in ANTs. A heatmap of Spearman correlation coefficients further supported the coordinated interplay among *S. flexneri* C.11 colonization, ERBB3 expression, and γH2AX in CRC (Figure S6E). Collectively, these findings demonstrated that *S. flexneri* C.11 promoted DNA damage and activated ERBB3 to play a pivotal role in CRC initiation and progression.

### Clinical diagnostic potential of *S.* *flexneri* C.11 and its metabolites

**Figure 8.**
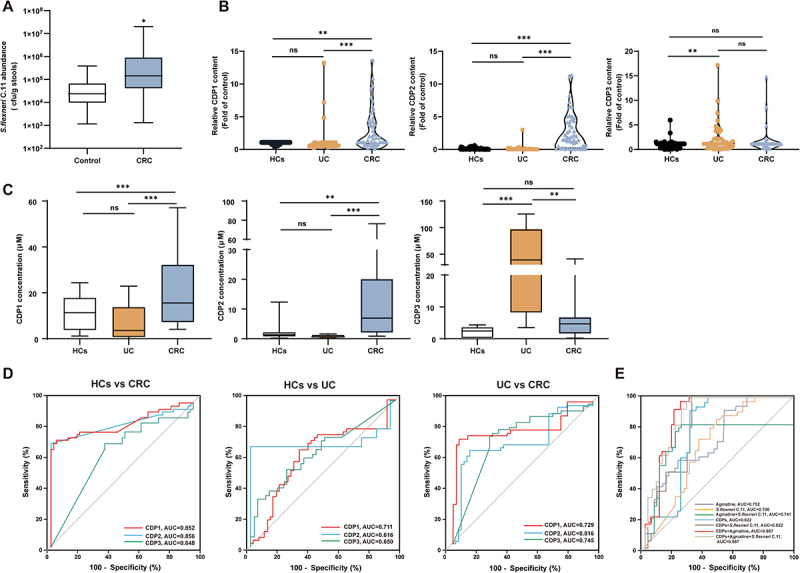
ROC characterization of identified metabolite markers in feces to UC, CRC patients and HCs.

After clarifying the carcinogenic effects of *S. flexneri* C.11 and CDPs, we performed a clinical sample regression analysis to further investigate their diagnostic potential and focused on assessing the changes in the abundance of *S. flexneri* C.11 and CDPs in fecal samples from UC and CRC patients compared to those from HCs. For *S. flexneri* C.11, we designed specific primers targeting its signature sequences and employed RT-qPCR to detect its relative abundance in fecal samples (Figure S7). The results revealed a significant increase in the abundance of *S. flexneri* C.11 in CRC patients compared to that in the normal control group (Figure 8(A)). Additionally, we analyzed CDP levels in fecal samples (Figure 8(B,C)). CRC patients showed significantly elevated CDP1 and CDP2 levels. In healthy individuals, CDP3 remained < 5 μM, while CDP1 and CDP2 ranged from 1.19–24.4 μM and 0.47–3.04 μM, respectively. Notably, CRC patients exhibited markedly increased CDP1 (6.3–57.09 μM) and CDP2 (1.09–76.28 μM), while CDP3 remained lower (0.19–40.9 μM). Western blotting assays further demonstrated that CDP1 induced significant DNA damage at 100 μM (*p* < 0.01), whereas CDP2 and CDP3 exhibited genotoxic effects at 50 μM (*p* < 0.05), as shown in Figure S5. These findings suggest that physiological CDP concentrations in healthy individuals are insufficient to trigger DNA damage.

(A) Targeted quantification of *S. flexneri* C.11 in HCs and CRC patients. (B – C) Targeted quantification of genotoxic metabolites in HCs, UC patients, and CRC patients. (D) ROC curve analysis of genotoxic metabolites. (E) ROC curve for multi-indicator joint diagnosis. Data, mean ± SEM. *p < 0.05, **p < 0.01, ***p < 0.001; NS, not significant.

To further evaluate the diagnostic potential of *S. flexneri* C.11 and its metabolites, a ROC analysis was conducted to assess the sensitivity and specificity of these biomarkers for distinguishing patients with CRC from normal controls (Figure 8(D)). Except for CDP3, the AUC values were greater than 0.7. Among these metabolites, CDP2 had the highest AUC (AUC = 0.856). In addition, combinations of markers identified using *S. flexneri* C.11, agmatine, and CDPs significantly improved the diagnostic accuracy for CRC (Figure 8(E)), with the AUC increasing to 0.887, implying that *S. flexneri* C.11 and its metabolites may have significant diagnostic value in CRC.

## Discussion

In this study, we observed changes in the metabolic profiles of patients with CRC. The differential metabolites identified, including tryptophan, methionine, L-isoleucine, and agmatine, are closely associated with the intestinal flora and play pivotal roles in maintaining the immune balance in the mammalian gut.^18,22,30–33^ Among these metabolites, agmatine attracted our attention the most. A significant enrichment of agmatine was observed in the feces of patients with CRC, while the endogenous content of agmatine was low and produced mainly by the metabolism of the gut microbiota.^32^ Agmatine has been linked to *E. coli* resistance through arginine decarboxylase-mediated arginine metabolism in acidic conditions *in vivo*.^33^ Studies have shown that agmatine derived from gut bacteria triggers inflammation and promotes the occurrence of colorectal tumors by activating the Wnt signaling pathway.^34^ Agmatine has recently been associated with *Bacteroidetes* and can act as an FXR agonist to promote polycystic ovarian syndrome in female mice.^35^ We observed that agmatine concentrations in normal human fecal samples ranged from 0.13 to 275 ng/mL, whereas in fecal samples from patients with CRC, the agmatine concentration was as high as 1,718 ng/mL. However, we also examined DNA damage in the IEC-6 cell model and found that the concentration of agmatine required to induce cellular DNA damage far exceeded physiological levels. Hence, we hypothesized that specific strains capable of producing agmatine are present in the intestines of patients with CRC and possess the ability to induce DNA damage, thereby facilitating CRC development.

Gut microbes serve as a potential source of DNA mutations, and the accumulation of such damage disrupts host genome stability and potentially accelerates mutations in premalignant and cancer cells.^36,37^ While microbial sequencing in CRC samples has been widely reported, it primarily elucidates the relationship between bacteria and metabolites. However, owing to the complexity of the gut microbiota and the limitations of metagenomic algorithms, strains identified through sequencing may be difficult to obtain or culture. To overcome these challenges, we employed a metabolite-oriented approach to isolate and identify agmatine-producing gut bacteria. Subsequently, whole – genome sequencing and the verification of DNA damage were performed.

As in human cells, the identified bacterial strains in *C. elegans* can cause DNA damage, triggering the upregulation of *CEP-1* and subsequent initiation of a DNA repair mechanism.^38^ Whole – genome sequencing and serological analyses revealed that the agmatine – producing strains were *S. flexneri* 2b and lacked the pks^+^ virulence island gene, which may be an unreported carcinogenic *S. flexneri*. *S. flexneri*, an opportunistic pathogen of *Shigella* bacteria, possesses various virulence factors allowing it to invade, replicate within, and evade host immune responses through intercellular spread in human gut epithelial cells.^39,40^ For example, the well-known bacteria *enterotoxigenic E. coli* has been reported to contain the locus of enterocyte effacement pathogenicity island, producing the *Shiga toxins*, is associated with severe illnesses like bloody diarrhea and HUS.^41^ However, our study revealed that the colonization of *S. flexneri* C.11, despite lacking the virulence island gene, similarly caused intestinal inflammation in pseudo-sterile mice and facilitated the transformation of inflammation to cancer in the colon.

To the best of our knowledge, this is the first report on the isolation of small-molecule metabolite CDPs from *S. flexneri*. CDP-derived natural products with 2,5-diketopiperazine scaffolds represent a significant category of secondary metabolites found in various organisms, such as fungi, bacteria, and plants. CDPs exhibit diverse bioactivities, including antibacterial, antiviral, virulence factor, and immunosuppressive activities.^42^ Notably, CDPs are produced by various pathogenic bacteria such as *Vibrio vulnificus*,^43,44^ where they regulate virulence and biofilm formation, profoundly influencing host-pathogen interactions and suggesting its potential role as virulence factors. For instance, cyclo(Phe-Pro) has been reported to induce DNA damage in mammalian cells through the generation of reactive oxygen species.^43^ In this study, sustained exposure to CDPs triggered DNA double-strand breaks, leading to dramatic alterations in cellular morphology, proliferation, migration, anchorage-independent growth, and cell cycle progression, ultimately driving malignant transformation. *In vivo* experiments further revealed that CDPs accelerated intestinal tumorigenesis in colitis model mice. It is well-established that CDP biosynthesis is primarily catalyzed by cyclodipeptide synthases (CDPSs) and nonribosomal peptide synthetases (NRPSs).^45^ Whole-genome analysis of *S. flexneri* C.11 identified the presence of NRPS gene clusters, suggesting a potential NRPS-dependent pathway for CDP synthesis in *S. flexneri* C.11. However, the direct involvement of NRPS genes in CDP biosynthesis requires further validation through targeted gene knockout and metabolomic profiling experiments.

Our findings demonstrate that *S. flexneri* C.11 and CDPs were significantly enriched in fecal samples from CRC patients. Diagnostic performance analysis revealed that both *S. flexneri* C.11 and CDPs exhibited AUC values exceeding 0.7. Notably, the combination analysis of *S. flexneri* C.11 abundance with CDPs levels achieved a markedly improved AUC of 0.887, underscoring its potential as noninvasive diagnostic tools for early CRC detection. However, several limitations must be addressed for clinical implementation, such as geographical variations in gut microbiota composition, driven by dietary habits, antibiotic exposure, or environmental factors, may limit the generalizability of *S. flexneri* C.11 as a universal biomarker. Multi – center studies across diverse populations are needed to validate its prevalence and diagnostic efficacy. In addition, host-specific factors including drug metabolism and microbial-host metabolic interactions could alter metabolite levels, requiring standardized detection protocols. Therefore, improving standardized detection methods in the future and combining these biomarkers with existing screening strategies is crucial for improving early detection of CRC.

ERBB3 is a member of the ERBB receptor tyrosine kinase family and has been found to be overexpressed in breast cancer, ovarian cancer, lung cancer, and CRC.^46–49^ Nrg1 serves as the principal ligand for the ERBB receptor family and predominantly interacts with ERBB3 receptors located on the cell membrane.^50^ The binding of Nrg1 to ERBB3 induces a conformational alteration, facilitating its heterodimerization with other ERBB family receptors (EGFR, ERBB2, and ERBB4). This heterodimerization promotes the autophosphorylation of the intracellular tyrosine kinase domain, initiating a signaling cascade. Our study confirmed that *S. flexneri* C.11 increased the expression of ERBB3 and Nrg1. *S. flexneri* C.11 and its metabolites enhanced the binding of ERBB3 to Nrg1, activating the PI3K-AKT and mTOR pathways, which subsequently promoted the malignant transformation of normal intestinal epithelial cells and progression of intestinal tumors in mice. Therefore, inhibiting the PI3K-AKT signaling pathway could be a promising therapeutic strategy for CRC, particularly in cases involving abundant *S. flexneri*, by targeting Nrg1, ERBB3, and their downstream signaling pathways.

**Figure 9.**
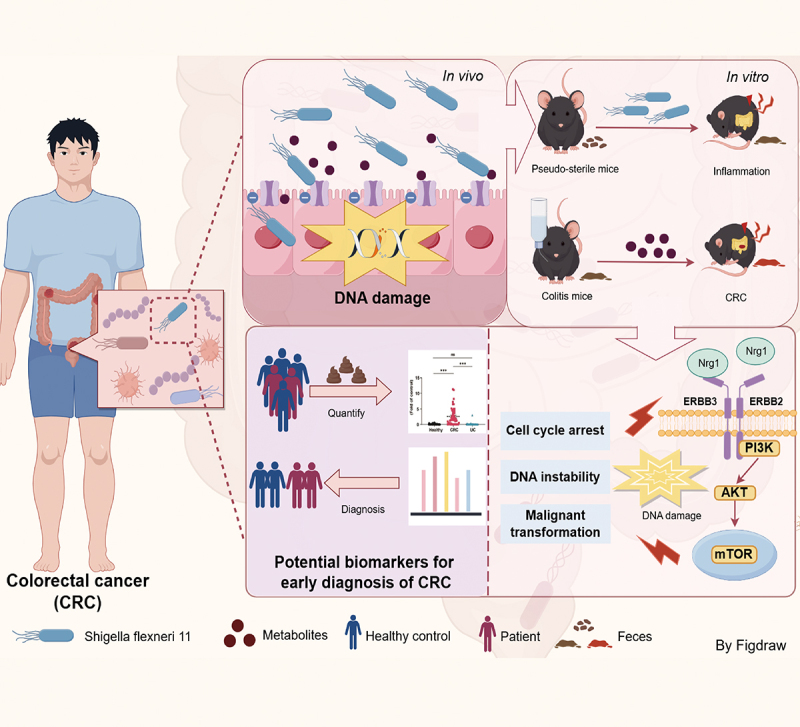
Mechanism and application of *S. flexneri* C.11 and its genotoxic in promoting CRC development in mice via DNA damage and activation of ERBB3/Nrg1/PI3K/AKT/mTOR signaling pathway.

In conclusion, this study unveils an unreported pathogenic strain of *S. flexneri* and identifies CDPs, a class of small-molecule gene toxins produced by *S. flexneri* C.11, which distinguish it from pks^+^-carrying *E. coli*. In addition, *S. flexneri* C.11 exhibited DNA-damaging activity and acted on the ERBB3 target, further activating the cascade pathway, increasing DNA damage in mice, and exacerbating colon tumor formation (Figure 9). Overall, this study reveals new microbiota-derived genotoxins and their potential impact on gut physiology and tumor risk, offering novel insights and strategies for CRC prevention and intervention.

## Supplementary Material

Informed_Consent_Document clean.docx

Table S5.csv

Supplementary_Materials_clean.docx

## Data Availability

All data relevant to the study are included in the article or uploaded as supplementary information. The metabolomics data that support the findings of this study are openly available in the National Genomics Data Center database: Bioproject PRJCA038115. The whole genome sequencing data of *S. flexneri* are available in the NCBI database: BioProject accession number PRJNA1245060. Raw data not included therein can be obtained with the consent of the corresponding author.

## Abbreviations

AFP: alpha-fetoprotein
ANTs: adjacent normal tissues
AOM: azoxymethane
AUC: area under the curve
*C.* *elegans*: *Caenorhabditis elegans*
CEA: carcinoembryonic antigen
CDPs: cyclodipeptides
CDPSs: cyclodipeptide synthases
CDP1: cyclo (Pro-Leu)
CDP2: cyclo (Phe-Pro)
CDP3: cyclo (Pro-Val)
CT-DNA: calf thymus DNA
COAD: colon adenocarcinoma
CRC: colorectal cancer
DSS: dextran sodium sulfate
*E. coli* K12: *Escherichia coli* K12
EGFR: epidermal growth factor receptor
FISH: fluorescence in situ hybridization
GNPS: Global Natural Product Social Molecular Networking
GTEx: Genotype-Tissue Expression
GO: gene ontology
HCs: healthy controls
H&E: hematoxylin-eosin
IBD: inflammatory bowel disease
KEGG: Kyoto Encyclopedia of Genes and Genomes
NRPSs: nonribosomal peptide synthetases
Nrg1: neuregulin 1
PBS: phosphate-buffered saline
PCNA: proliferating cell nuclear antigen
PCoA: principal coordinate analysis
PLS-DA: partial least squares discriminant analysis
QC: quality control
READ: rectal adenocarcinoma
*S. flexneri*: *Shigella flexneri*
ROC: receiver operating characteristic
SEM: standard error of the mean
TCGA: The Cancer Genome Atlas
VIP: variable importance in projection
UC: ulcerative colitis.
