# Supplementary material for: A new gut pathogenic bacteria and its metabolites promote colorectal cancer development and act as non-invasive early diagnostic biomarkers
Source: Gut Microbes. 2025 Sep 5;17(1):2555446. doi: 10.1080/19490976.2025.2555446 (PMC12416176; doi:10.1080/19490976.2025.2555446)
Supplement: Informed_Consent_Document clean.docx [file KGMI_A_2555446_SM8643.docx]

**Informed Consent Document**

Dear patient,

This Informed Consent Form is intended to seek your consent for the collection and preservation of biological samples and health-related information by the Anorectal Center of Nanjing Hospital of Traditional Chinese Medicine for the purpose of conducting medical teaching and research.

Please read the following contents carefully to make your decision. This decision is entirely your voluntary choice. When a representative from Nanjing Hospital of Traditional Chinese Medicine (your attending physician or a designated hospital staff member) discusses this Informed Consent Form with you, you can ask them to explain any parts you do not understand.

**Section 1: Basic Information about the Biobank**

To regulate medical research and promote the progress of biotechnological advancements, the biobank is established by Nanjing Hospital of Traditional Chinese Medicine as an institution to standardize and systematize the collection, preservation, utilization, and external provision of human biological samples. The purpose of the biobank is to provide standardized disease research materials for biomedical education and research, promote advancements in disease diagnosis and treatment, improve public health levels, and benefit all people, including you.

The biobank will operate under the supervision and guidance of relevant administrative departments, comply with current laws, regulations, and policies, adhere to recognized ethical guidelines, conform to the provisions on human genetic resources, respect the conventions and practices of biomedical research, and standardize the work related to the collection, preservation, utilization, external provision, and donor protection of biological samples.

The Ethics Committee of Nanjing Hospital of Traditional Chinese Medicine will strictly review the work of the biobank according to relevant regulations to protect your rights and ensure that the biobank’s operations comply with relevant laws and ethical standards.

**Section 2: Potential Discomfort, Risks, and Protective Measures for Biological Sample Donation**

The donated samples primarily come from your bodily fluids and tissue specimens that have no clinical value. Specifically, during routine medical treatment, the hospital will use your bodily fluids (e.g., blood, urine) and/or surgically removed tissue specimens for necessary medical examinations and pathological diagnoses. After testing, any remaining specimens may be preserved by the biobank if you agree to donate them, rather than being destroyed. For bodily fluids, appropriate amounts will be retained and saved during necessary medical tests. The collection of these samples will not harm your health or cause any adverse effects.

**Section 3: Common Benefits and Significance of Donation**

The collection and preservation of biological samples will not require any payment from you. Scientific research primarily aims to advance science and technology and does not generate direct economic benefits or welfare. If any patent rights or commercial interests arise from the research results, all rights will not be related to you. You cannot obtain any direct benefits from donating biological samples, including direct economic compensation or exemptions from diagnostic and treatment fees.

Your contribution and that of other donors will promote advancements in medical technology, leading to more effective methods for disease diagnosis and treatment. This will benefit you and other patients with similar conditions, which constitutes the common interest of you and other donors.

Currently, it is unknown how your information or samples will be used in specific research, nor can it be predicted whether the research results will have any impact on your health or that of your family or ethnic group. If significant health issues related to you are discovered during the research, the biobank will notify you appropriately in accordance with national ethical guidelines and procedures.

**Section 4: Confidentiality Scope and Measures for Donor Information**

Medical research focuses on studying diseases and does not involve your personal privacy or identifiable information. We will strictly protect the privacy and personal information of every donor by implementing the following measures:

1. Establish confidentiality measures and systems for donor information security (personal information protection system). All collected samples will be managed anonymously (through coding and/or the removal of all identifiable information).
2. Securely store samples and data, and set access permissions for all samples and/or data in the biobank.
3. When samples or research materials (samples transferred for research purposes) are provided to other researchers or institutions, no research institution or individual will obtain your personal identity or private information.
4. Research data may be publicly published or disclosed, but your name or identifiable personal information will not be disclosed.

**Section 5: Donor’s Right to Autonomy**

You have the right to refuse to sign this Informed Consent Form. Refusing to sign this document will not affect any of your rights or interests, nor will it result in discrimination, differential treatment, or impact your normal diagnosis and treatment. You may withdraw from the donation at any time without conditions, requiring the biobank to cease the collection, use, and preservation of biological samples sourced from you.

**Informed Consent Declaration**

1. I have read and understood all the contents of this Informed Consent Form.
2. I have had the opportunity to ask questions, and all my questions have been answered.
3. I understand that participation in this activity is entirely voluntary, and I have the right to withdraw at any time without conditions. My medical treatment and rights will not be affected as a result.
4. I am aware that signing this Informed Consent Form does not exempt me from any fees or responsibilities.
5. I understand that if I have any further questions after signing this Informed Consent Form, I can consult the staff of the Ethics Committee of Nanjing Hospital of Traditional Chinese Medicine.

**Voluntary Donation Declaration**

I hereby voluntarily donate my biological samples and information to the biobank of Nanjing Hospital of Traditional Chinese Medicine. I agree that the donated samples and information may be used for all medical research purposes to contribute to the early conquest of diseases and the treatment of patients.

**Donor’s Signature:** __________________________
**Date:** ____________ (Year) ____________ (Month) ____________ (Day)

*(Note: If the donor lacks full legal capacity or has limited legal capacity, the legal guardian must sign and provide the date.)*

**Legal Guardian’s (or Custodian’s) Signature:** __________________________
*(Relationship to the donor: ____________)*
**Date:** ____________ (Year) ____________ (Month) ____________ (Day)

**Declaration of the Informed Consent Informant**

I have accurately explained all contents of the Informed Consent Form to the donor and answered all questions raised by the donor.

**Informed Consent Informant’s Signature:** __________________________
**Date:** ____________ (Year) ____________ (Month) ____________ (Day)
