# Supplementary material for: A new gut pathogenic bacteria and its metabolites promote colorectal cancer development and act as non-invasive early diagnostic biomarkers
Source: Gut Microbes. 2025 Sep 5;17(1):2555446. doi: 10.1080/19490976.2025.2555446 (PMC12416176; doi:10.1080/19490976.2025.2555446)
Supplement: Supplementary_Materials_clean.docx [file KGMI_A_2555446_SM8641.docx]

**Supplementary Materials for**

**A new gut pathogenic bacteria and its** **metabolites promote colorectal cancer development** **and** **act as non-invasive early diagnostic biomarkers**

Rui Zhang^&1^, Mingxiao Li^&1^, Hui Tan^1^, Jianyao Liu^1^, Lu Wang^2^, Wenling Dai^*1^, Zhimin Fan^*2^, Ji-Hua Liu^*1,3^

*^1^Jiangsu Key Laboratory of TCM Evaluation and Translational Research, School of Traditional Chinese Pharmacy, China Pharmaceutical University, Nanjing, Jiangsu 211198, China*

*^2^Jiangsu Clinical Innovation Center for Anorectal Diseases of T.C.M, Nanjing Hospital of Chinese Medicine, Nanjing 210022, P.R. China*

*^3^State Key Laboratory of Natural Medicines, School of Traditional Chinese Pharmacy, China Pharmaceutical University, Nanjing, Jiangsu, 210009, China*

^&^These authors contributed equally to this work and should be considered co-first authors.

* Corresponding author: Jihua Liu, Wenling Dai, Zhimin Fan,

**Table of contents**

**Supplementary** **materials and methods**

**Supplementary figures (S1-S7)**

**Supplementary tables (S1-S5)**

**SUPPLEMENTARY MATERIALS AND METHODS**

**Cell treatment**

IEC-6 cells were purchased from ATCC and cultured in DMEM containing 10% v/v fetal bovine serum (FBS) at 37℃ in a humidified atmosphere containing 5% CO_2_. For Western blot and comet assay experiments, IEC-6 cells were plated at 5 × 10^6^/well in 6 well tissue culture plates, while for immunofluorescence experiments, IEC-6 cells were plated on coverslips in 24 well plates at 1 ×10^5^/well. Both of these were allowed to adhere overnight. Cells were infected with *E. coli* K12 and *S.flexneri* C.11 (1 × 10^8^ cfu/mL) for 4 h at 37℃. Cells were washed with PBS and fixed immediately for γH2AX staining.

**Comet assay**

The comet assay was performed with the Trevigen Comet Assay kit (Trevigen) according to the manufacturer protocol. Briefly, IEC-6 cells were collected in PBS to a concentration of 1 × 10^5^ cells/mL, mixed with 37℃ 1% low-melting agarose and loaded on 2 well CometSlides. CometSlides were placed in the pre-chilled lysis solution at 4℃ for 60 min, and then incubated in alkaline unwinding solution at room temperature for 20 min in the dark. CometSlides were transferred to a pre-chilled fresh alkaline electrophoresis solution and subjected to electrophoresis using the CometAssay Electrophoresis System II (Trevigen) for 30 min (21 V). Slides were washed twice in ddH_2_O for 5 min and 70% ethanol for 5 min. DNA was stained with 50 µL DAPI in a light-protected setting for 30 min and visualized using the confocal laser scanning microscope (CLSM, Carl Zeiss LSM 700).

**Immunofluorescence detection of γH2AX in cell lines**

The IEC-6 cells were washed with PBS twice and then blocked with 10% normal donkey serum containing 0.3% Triton X-100. After incubation with the γH2AX rabbit monoclonal antibody overnight at 4℃, the cells were washed with PBS and incubated in the secondary Alexa Fluor 488 goat anti-rabbit antibody for 2 h at room temperature. Before taking images, cells were washed again with PBS and mounted with DAPI for 10 min. Fluorescent images were observed with a confocal laser scanning microscope (CLSM, LSM 700, Zeiss, Oberkochen, Germany) and processed using the ZEN imaging software.

**The *C. elegans* maintenance and lifespan statistics**

*C. elegans* were maintained on nematode growth medium (NGM) plate with *Escherichia coli* OP50 as standard food. About 150 L4 stage (day 0 for lifespan assays) *C. elegans* were transferred to plates with *E. coli* K12 or *S. flexneri* C.11 (10^8^ cfu) at 20℃. 5-Fluoro-2’-deoxyuridine was used to prevent reproduction. *C. elegans* were transferred to new plates and counted every other day. *C. elegans* that did not move when gently prodded were scored as dead. The *C. elegans* crawled off the plate were censored.

**Microscopy and DAPI staining of *C. elegans***

For staining with DAPI, 30 *C. elegans* were transferred to a 1.5 mL Eppendorf tube filled with 20 μL of cold methanol and kept at –20℃ for 5 min. Methanol was aspirated, and 20 μL of S-basal was added and pipetted up and down to separate bacteria stuck to *C. elegans*. The bacteria suspended in S-basal were removed. Finally, 10 μL of S-basal with 0.5 μL of DAPI solution (×200) was added to the *C. elegans* pellet. After 5 min of incubation with DAPI, the *C. elegans* corpses were mounted on a microscopic agarose pad. Fluorescent images were observed with a confocal laser scanning microscope and processed using the ZEN imaging software.

**Analysis of *C. elegans* epithelial junctions**

After 72 h of exposure to pathogenic bacteria or control bacteria (*E. coli* OP50), *C. elegans* were washed down with M9 buffer and centrifuged at 1000 rpm for 5 min. 2% agarose was prepared and dissolved using a microwave oven. The agarose was dropped on the slide, and the other slide was covered with adhesive pad. After recovery, fluorescent blue dye (5%) was added. After 3 h, the *C. elegans* were recovered by centrifugation and rinsed repeatedly with M9 buffer until the washing solution was colorless.

**Gut microbiota analysis**

Total genomic DNA from samples was extracted using CTAB/SDS method. DNA concentration and purity were monitored on 1% agarose gels. According to the concentration, DNA was diluted to 1 ng/µL using sterile water. 16S rRNA genes of distinct regions were amplified using forward primer 515F-50 -GTGCCAGCMGCCGCGG-30 and reverse primer 907R-50 -CCGTCAATTCMTTTRAGTTT-30. All PCR reactions were carried out with 15 µL of Phusion High-Fidelity PCR Master Mix (New England Biolabs); 0.2 µM of forward and reverse primers, and about 10 ng template DNA. Thermal cycling consisted of initial denaturation at 98℃ for 1 min, followed by 30 cycles of denaturation at 98℃ for 10 s, annealing at 50℃ for 30 s, and elongation at 72℃ for 30 s, and a final extension at 72°C for 5 min 72℃ for 5 min. Mix an equal volume of 1 × loading buffer (contained SYB green) with PCR products and subjected to electrophoresis on 2% agarose gel for detection. PCR products were mixed in equidensity ratios. Then, mixture PCR products was purified with Qiagen Gel Extraction Kit (Qiagen, Germany). Sequencing libraries were generated using TruSeq DNA PCR-Free Sample Preparation Kit (Illumina, USA) following manufacturer's recommendations and index codes were added. The library quality was assessed on the Qubit@ 2.0 Fluorometer (Thermo Scientific) and Agilent Bioanalyzer 2100 system. At last, the library was sequenced on an Illumina NovaSeq platform and 250 bp paired-end reads were generated.

Alpha diversity is applied in analyzing complexity of species diversity including observed-species, chao1 and shannon. All these indices in our samples were calculated with QIIME (Version 1.7.0) and displayed with R software (Version 2.15.3). We employed the online tool LefSe to explore significantly affected bacteria based on genus abundance. The threshold for the logarithmic liner discriminant analysis (LDA) score was set at 2, and other parameters were set as default.

**RNA Sequencing**

Total amount and integrity of RNA were assessed using the RNA Nano 6000 Assay Kit of the Bioanalyzer 2100 system (Agilent Technologies, CA, USA). Total RNA was used as input material for the RNA sample preparations. Briefly, mRNA was purified from total RNA by using poly-T oligo-attached magnetic beads. Fragmentation was carried out using divalent cations under elevated temperature in First Strand Synthesis Reaction Buffer(5 ×). First strand cDNA was synthesized using random hexamer primer and M-MuLV Reverse Transcriptase, then the RNA was degraded with RNase-free water. Second strand cDNA synthesis was subsequently performed using DNA Polymerase I and dNTP. Remaining overhangs were converted into blunt ends via exonuclease/polymerase activities. After adenylation of 3’ ends of DNA fragments, Adaptor with hairpin loop structure were ligated to prepare for hybridization. In order to select cDNA fragments of preferentially 370–420 bp in length, the library fragments were purified with AMPure XP system (Beckman Coulter, Beverly, USA). Then after PCR amplification, the PCR product was purified by AMPure XP beads, and the library was finally obtained. In order to ensure the quality of the library, the library was assessed. After the construction of the library, the library was initially quantified by Qubit2.0 Fluorometer, then diluted to 1.5 ng/uL, and the insert size of the library is detected by Agilent 2100 bioanalyzer. After insert size meets the expectation, RT-qPCR was used to accurately quantify the effective concentration of the library (the effective concentration of the library is higher than that of 2 nM) to ensure the quality of the library.

**Quantification of *S. flexneri* C.11 in clinical fecal sample**

Accurate quantitation of the number of cells of individual bacterial species is needed for understanding the role of *S. flexneri* C.11 in the progression of colitis to CRC. This study described a real-time PCR method for strain-specific identification and quantification of *S. flexneri* C.11 in human clinical feces samples. The primers were selected following an alignment of the entire genome sequence and proved to be specific for *S. flexneri* C.11. The assay could detect approximately 10^2^ cfu/mL of *S. flexneri* C.11. Measurements of analytical sensitivity determined with spiked control strain *E. coli* K12 indicated that the consistent limit of detection was approximately 10^3^ cfu/g of spiked samples and about 10^3^ cfu/g of clinical cecal samples. The results indicate that the SYBR Green real time PCR assay is a useful diagnostic tool for quick, high throughput and reliable routine screening of *S. flexneri* C.11.

**Bacterial strains and DNA isolation**

The *E. coli* K12 and bacterial *S. flexneri* C.11 were cultured in grown in Luria Bertani (LB) nutrient broth at 37℃ for 16 h, then subjected to DNA extraction for SYBR Green I real-time PCR and melting curve analysis. Fecal DNA extraction was carried out using TIANamp Stool DNA Kit (TIANGEN, Beijing) and the manual method. The DNA concentration and purity were measured using NaCDrop 2000 spectrophotometer (Thermo Fisher Scientific, Waltham, MA, USA) and stored at –20℃ before use.

**Primer design**

Genomic testing of *S. flexneri* C.11 was carried out to illuminate the characteristics of *S. flexneri* C.11 and obtain its genetic information and virulence factor information. GenomeComp1.3 software, which provides a graphic user interface (GUI), is a software for DNA sequence comparisons. First, we used this software to compare the two sequences (*S. flexneri* C.11 and *E. coli* K12) and to identify their differential sequences. Then, to confirm these results, we used the programs BLASTN, BLASTP, and BLASTX (http://www.ncbi.nlm.nih.gov/) to locate the sequence similarities in the nucleotide and protein databases. Primers were designed based on the DNA sequence ranging from the 4853 to 6189 nucleotide positions of 1336 bp DNA fragment. Primers were designed using the Primer Express 2.0 software (Applied Biosystems, Foster City, USA), with the software's default settings. Candidate primers sequences were examined for specificity following BLAST (http://www.ncbi.nlm.nih.gov/blast/) nucleotide sequence database searches for DNA sequences. Subsequently designed on Primer 5.0 and synthesized by Sango Biotech Co. (Shanghai, China). Primers as follow:

(F) 5′–CTTTGCAACAGTGCCACTCA‐3′ (R) 5′–CCATTCTGTCGCGTAGCGT‐3′

**Standards for quantification**

A pure culture and a feces-based standard series of *S. flexneri* C.11 were obtained. For pure culture standards, a tenfold dilution series of the strain in LB was prepared. For feces-based standards, suitable amounts of *S. flexneri* C*.*11 dilutions were spiked to feces, resulting in standards that contained 10^8^ to 10^1^ cfu of *S. flexneri* C.11 per mL. DNA was extracted from 1 mL of these suspensions as described before, then RT-qPCR was performed to establish the standard curves. The precise number of cfu in the dilutions was obtained by the plate count method.

**SYBR Green real-time PCR protocols**

Each real-time PCR reaction included 60 ng of DNA, 2 × Talent qPCR PreMix (SYBR Green), 10 μM primer mixture and RNase free water. All reactions were detected under the following conditions: 95℃ for 5 min, 95℃ for 15 s, 54℃ for 20 s, and 72℃ for 15 s, a total of 40 cycles. Subsequently, fluorescence signals were collected at 95℃ for 15 s, 60℃ for 1 min, and 95℃ for 1 s. Then, use the QuantStudio Design&Analysis Software for the testing and result analysis. The 16s rRNA total bacterial DNA measured by qPCR was used to standardize target genes in fecal samples. All qPCR reactions were performed in duplicate. The relative number of *S. flexneri* C.11 in each fecal sample is determined by the 2^(-△Ct) method, using the 16s rRNA gene as the reference gene: △Ct = Ct *S. flexneri* C.11 gene-Ct 16S rRNA.

**Screening of DNA adduct using CT-DNA fishing coupled with HPLC-QTOF-MS**

Figure 4A illustrates the screening process for CT-DNA adducts utilizing HPLC-QTOF-MS in conjunction with ligand fishing techniques. A solution of CT-DNA at a concentration of 10 mg/mL was prepared and subsequently combined with the fermentation broth of *S. flexneri* C.11 in PBS solution (final CT-DNA concentration was 1 mg/mL), followed by 24 h incubation at 37℃ with gentle shaking. The sample was then subjected to 3 kd ultrafiltration (4℃, 5000 g, 30 min) with the filtrate discarded. This washing procedure was repeated twice with PBS buffer. The retentate in the upper chamber of the ultrafiltration tube was then collected and treated with 600 μL of 95% methanol containing 1% formic acid to facilitate dissociation of potential adducts from CT-DNA. A final ultrafiltration step was conducted, after which the collected filtrate was dried under a stream of nitrogen, reconstituted in 50 μL methanol, filtered, and analyzed by HPLC-QTOF-MS.

**Receiver operator characteristic analysis**

The diagnostic performance of *S. flexneri* C.11 and its metabolites was evaluated using the Xiantao Academic online platform (https://www.xiantao.love/products). Specifically, ROC analysis was used to assess the performance of the microbial biomarkers using the “pROC” package (1.18.0) in R (4.2.1). The results were shown by the “ggplot2” package (3.4.4)

**Fluorescence in situ hybridization**

The Cy3-TSA fluorescence probe (red signal) was synthesized by Boerfu Biotechnology Co., Ltd. (Wuhan, China). Specifically, the parpered tissue slices were baked at 50℃ for 2–3 h followed by deparaffinization. Antigen retrieval was performed by boiling slides in retrieval solution for 10–15 min depending on tissue fixation duration. After permeabilization, enzymatic digestion, washing, and dehydration, slides were air-dried at room temperature. Denatured/pre-annealed DNA probe (10 μL) was applied to denatured dehydrated specimens, covered with 18 × 18 mm coverslips, sealed with Parafilm, and hybridized in a humidified chamber at 37℃ overnight (15–17 h). Post-hybridization, coverslips were gently removed with a blade. Slides underwent three sequential 5 min washes in pre-warmed 50% formamide/2 × SSC (42–50℃), followed by three 5 min washes in pre-warmed 1 × SSC (42–50℃) before air-drying. DAPI staining and sealing were performed prior to fluorescence microscopic observation.

**Immunofluorescence detection of γH2AX and ERBB3 in clinical tissues**

The tissue sections were washed with PBS twice and then blocked with 10% normal donkey serum containing 0.3% Triton X-100. After incubation with the γH2AX and ERBB3 antibody overnight at 4℃, the cells were washed with PBS and incubated in the secondary Alexa Fluor 488 goat anti-rabbit antibody for 2 h at room temperature. Before taking images, tissue sections were washed again with PBS and mounted with DAPI for 10 min. Fluorescent images were observed with a confocal laser scanning microscope (CLSM, LSM 700, Zeiss, Oberkochen, Germany) and processed using the ZEN imaging software.

**Expression and prognostic analysis of ERBB3 in public databases**

We downloaded STAR-counts data and corresponding clinical information for COAD and READ tumors from the TCGA database (https://portal.gdc.cancer.gov) and GTEx database (https://gtexportal.org/home/datasets). We then extracted data in TPM format and performed normalization using the log2(TPM+1) transformation. After retaining samples that included both RNAseq data and clinical information, we ultimately selected 1275 COAD samples and 954 READ samples for further analysis. Statistical analysis was conducted using R software, version v4.0.3. Results were considered statistically significant when the *p*-value was less than 0.05. Detailed data can be found in Table S5.

Violin plots depicting ERBB3 expression across CRC stages were generated using the ggpubr package, with Wilcoxon rank-sum tests for statistical comparisons. Kaplan-Meier survival analysis was performed using the survminer and survival packages in R software to compare overall survival between ERBB3 low-expression and high-expression groups. Survival probabilities at each time point were calculated to visualize dynamic survival rate changes over time.

**Genome sequencing of *S. flecneri* C.11**

Genomic DNA of *S. flexneri* was extracted from samples using SDS/STE methods, followed by agarose gel electrophoresis to assess DNA purity and integrity. Sequencing was performed by Novogene Technology Co., Ltd. (Tianjin, China). Key procedures included: Large DNA fragments were first recovered using the BluePippin automated nucleic acid fragment recovery system, followed by end repair. Barcoding was performed using Oxford Nanopore Technologies’ EXPNBD104 kit via a PCR-free method. Fragment size was analyzed using the AATI automated capillary electrophoresis system, followed by equimolar pooling of samples. Adapter ligation was conducted with Oxford Nanopore Technologies’ SQK-LSK109 ligation kit to construct a 10 K library, which was sequenced on the Nanopore platform to generate the Nanopore 1D library. After passing quality control, libraries were loaded onto the Nanopore PromethION platform for sequencing, with loading volumes adjusted based on effective library concentrations and target sequencing data yield. Post-assembly analyses included genome component prediction (coding genes, non-coding RNAs, repeats, prophages, genomic islands, CRISPR systems). Coding sequences were functionally annotated against databases (KEGG, COG, pathogen-specific databases). Comparative genomic analyses against reference genomes encompassed synteny analysis, gene family construction, core/unique gene identification, and phylogenetic tree reconstruction.

**SUPPLEMENTARY FIGURES**


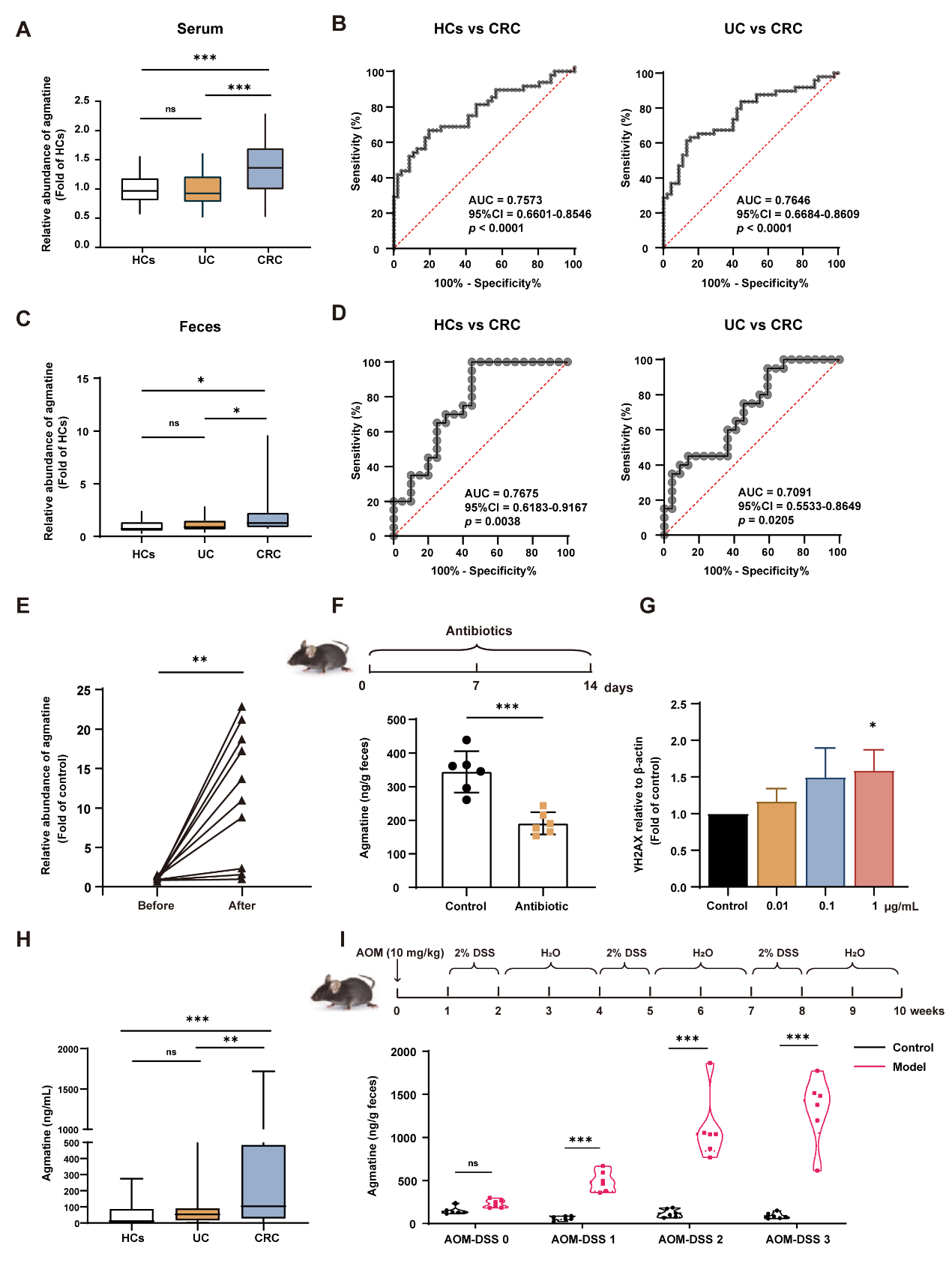


**Figure S1**

Bacterial metabolite agmatine was increased in CRC patients. (A) Relative abundance of agmatine in serum. (B) Receiver operating characteristic (ROC) analysis for agmatine discriminating CRC from healthy controls (HCs). (C) Relative abundance of agmatine in feces. (D) ROC analysis for agmatine discriminating CRC from UC. (E) Production of agmatine in an *in vitro* gut simulator inoculated with feces from CRC patients (n = 10). (F) Agmatine content in antibiotic treated mice (14 days) and control group (n = 6). (G) Effect of agmatine at concentrations of 0.01, 0.1, and 1 μg/mL on the expression level of γH2AX, a DNA damage marker. (H) Agmatine content in CRC patients. (I) Fecal levels of agmatine in AOM-DSS treated mice (n = 6). Data, mean ± SEM. **p<*0.05, ***p<*0.01, and ****p<*0.001 indicates significant differences compared to control.

**
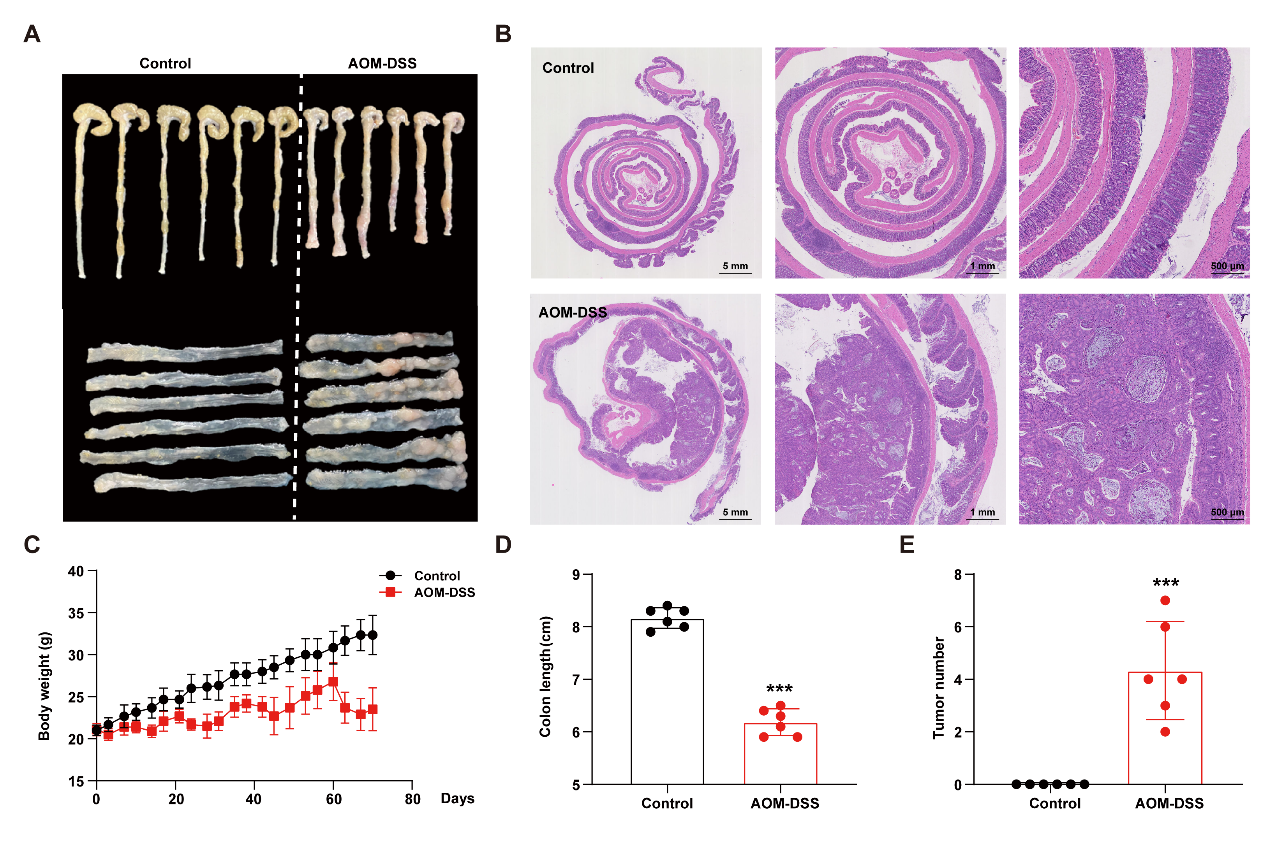
**

**Figure S2**

Colon tumors were formed spontaneously in AOM-DSS model animals (n = 6). (A) Macroscopic morphologies of colon sections in each group. (B) H&E-stained colon sections of mice in each group. (C) Body weight of mice in each group (D) Colon length of mice in each group. (E) Tumor number of mice in each group. Data, mean ± SEM. **p<*0.05, ***p<*0.01, and ****p<*0.001 indicates significant differences compared to control.

**
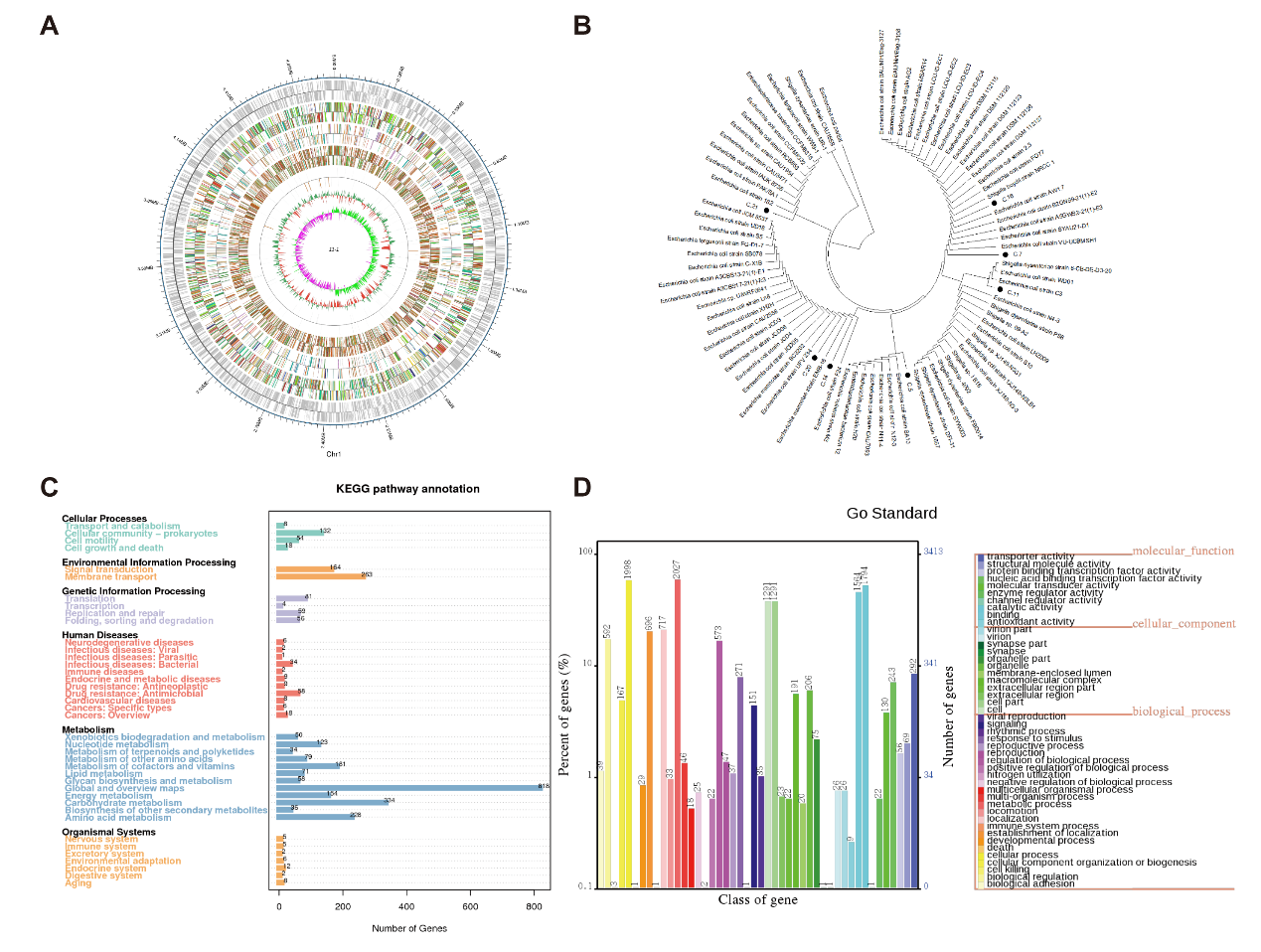
**

**Figure S3**

Whole genome sequencing and absolute quantification of *S. flexneri* C.11. (A) Genome-wide map of *S. flexneri* C.11. (B) GTDB-Tk database construction of strain *S. flexneri* phylogenetic tree results. (C) KEGG metabolic pathway classification map. (D) GO functional classification map.

**
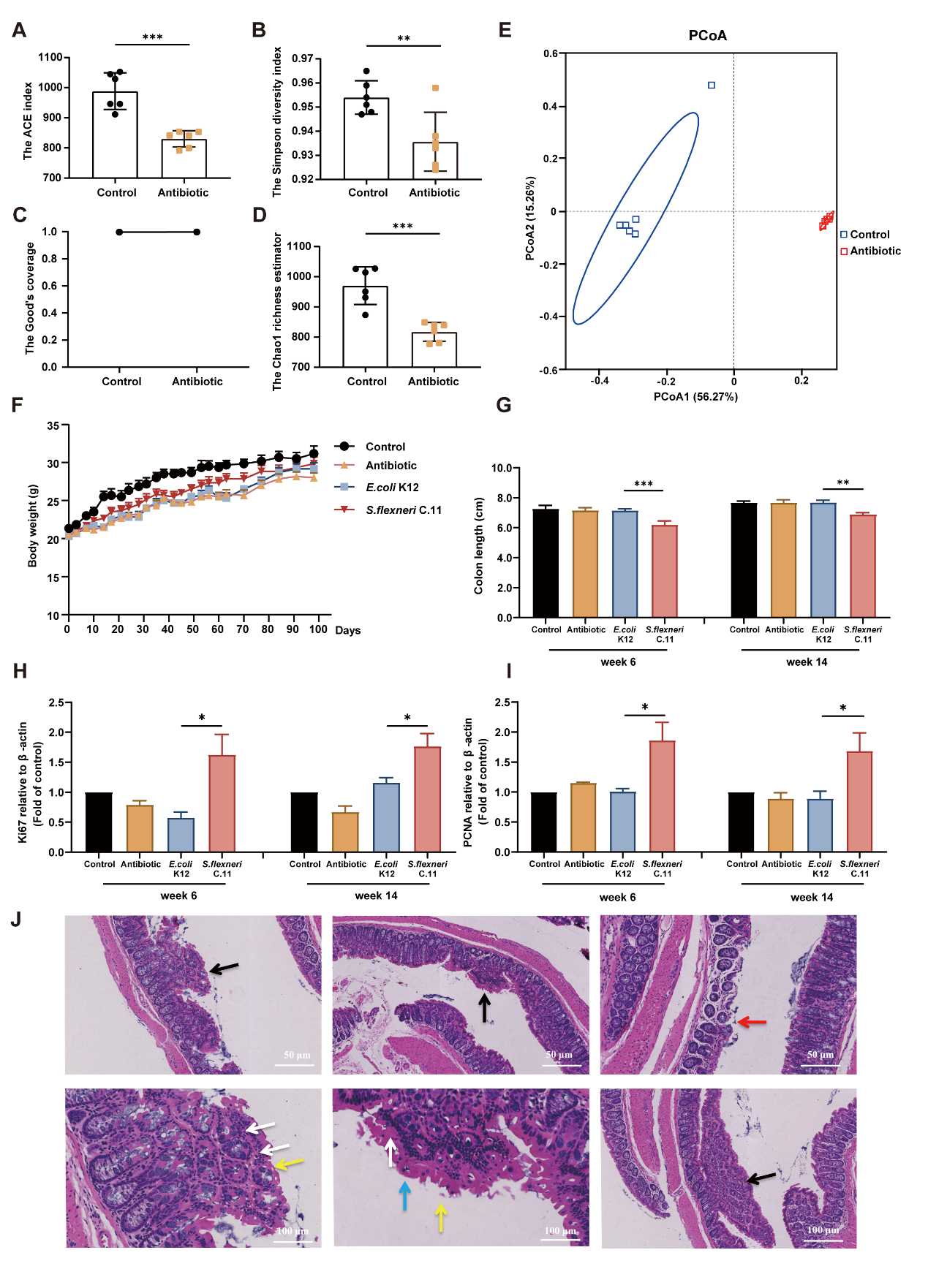
**

**Figure S4**

*S. flexneri* C.11 promotes colorectal tumorigenesis in mice (n = 6). Changes in gut microbiota diversity after antibiotic treatment (A) Abundance-based Coverage Estimator (ACE) index (B) Simpson index (C) Good's coverage (D) Chao1 richness estimator (E) Principal Coordinates Analysis (PCoA). (F) Body weight of each group (G) Colon length in each group (H-I) Western blot analysis of Ki67 and PCNA expression in mice treated with *S. flexneri* C.11 or *E. coli* K12 for 6 and 14 weeks. (J) Representative H&E staining images of intestinal tissues from *S. flexneri* C.11-treated mice. Data, mean ± SEM. **p<*0.05, ***p<*0.01, and ****p<*0.001 indicates significant differences compared to control.

**
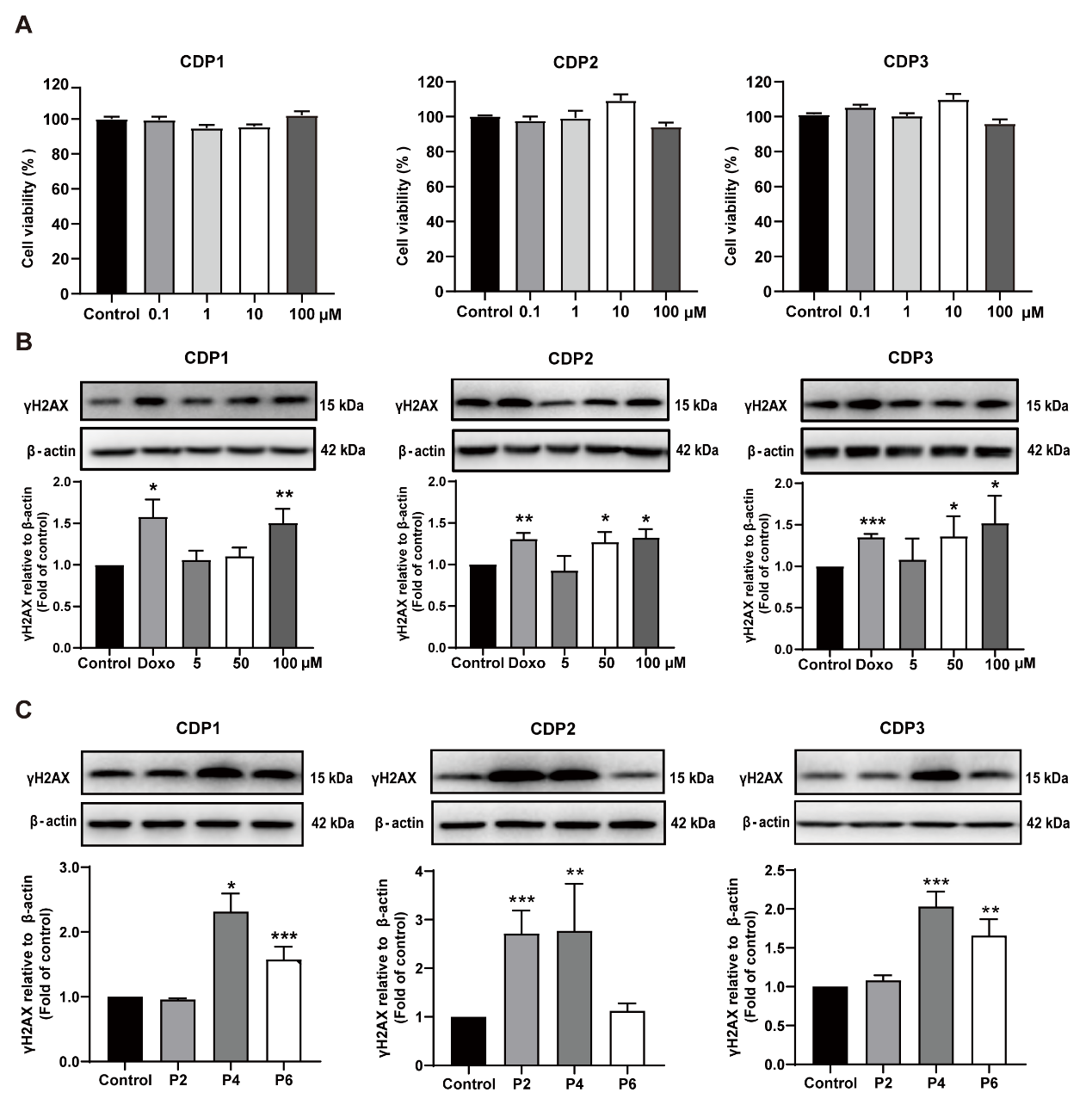
**

**Figure S5**

The direct effects of *S. flexneri* C.11 genotoxic metabolites on cells. (A) The effect of CDPs on cell viability. (B) DNA damage ability of CDPs at different concentrations (5, 50, 100 μM). (C) DNA damage in cells exposed to multiple passages of CDPs. Data, mean ± SEM. **p* < 0.05, ***p* < 0.01, ****p* < 0.001.


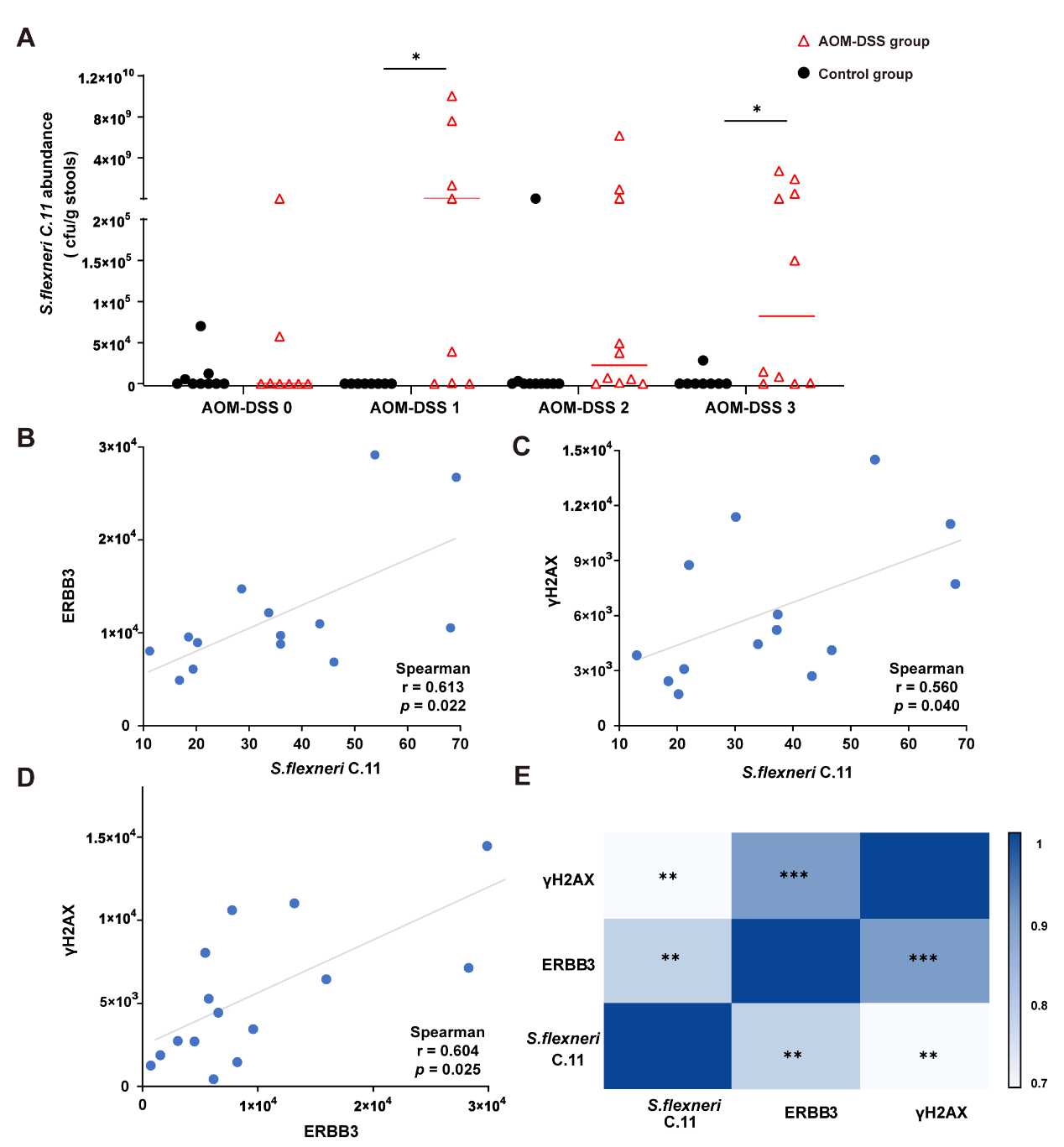


**Figure S6**

Clinical correlative validation of the *S. flexneri* C.11-ERBB3-DNA damage pathway in CRC. (A) Quantification of *S.flexneri* C.11 in AOM-DSS treated mice. (B) Correlation analysis of ERBB3 and *S. flexneri* C.11 in clinical tissue samples. (C) Correlation analysis of γH2AX and *S. flexneri* C.11 in clinical tissue samples. (D) Correlation analysis of γH2AX and ERBB3 in clinical tissue samples. (E) Correlation coefficient matrix diagram. Data, mean ± SEM. **p* < 0.05, ***p* < 0.01, ****p* < 0.001.


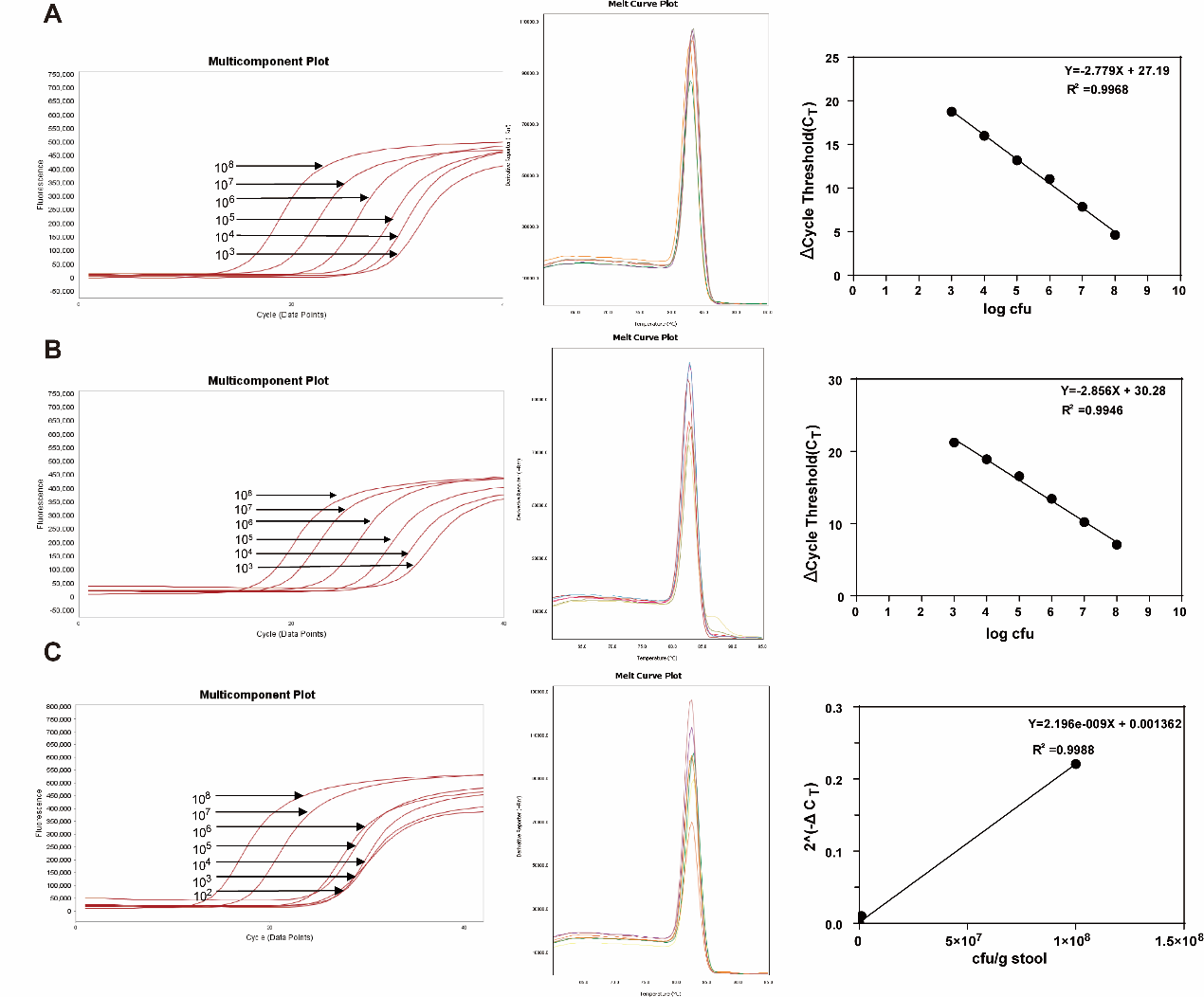


**Figure S7**

Amplification and melting curves and standard curves. (A) Real-time PCR amplification of *S. flexneri* C.11 genomic DNA. (B) Standard curve of *E. coli* K12 negative control samples. (C) Standard curve of clinical stool sample.

**SUPPLEMENTARY TABLES**

**Table S1**

Clinical characteristics of the subjects.

|  | Characteristics | HCs | UC | CRC |
| --- | --- | --- | --- | --- |
| Serum | Number | 46 | 45 | 49 |
|  | Male | 10 | 24 |  |
|  | Female | 36 | 21 |  |
|  | Age (years), mean ± SEM | 30.48 ± 6.38 | 43.51 ± 12.72 |  |
| Feces | Number | 20 | 22 | 20 |
|  | Male | 10 |  | 11 |
|  | Female | 10 |  | 9 |
|  | Age (years), mean ± SEM | 24.50 ± 1.19 |  | 60.80 ± 10.68 |
| Tissues | Number | 10 |  | 10 |
|  | Male | 4 |  | 4 |
|  | Female | 6 |  | 6 |
|  | Age (years), mean ± SEM | 71.4 ± 8.14 |  | 71.4 ± 8.14 |

**Table S2**

Key differential metabolites between the healthy, UC and CRC groups.

|  | Metabolites | m/z | Rt | p | FC | VIP | Gene |
| --- | --- | --- | --- | --- | --- | --- | --- |
| Serum Healthy vs CRC | LPC 18:2 | 519.3316 | 30.95 | 3.288E-02 | 1.36 | 1.17 | BCAT1, BCAT2, IARS, ACADSB |
| Serum Healthy vs CRC | PC(0:0/20:4) | 543.3328 | 31.69 | 2.786E-03 | 1.83 | 1.45 | DDC, IDO1, TPH1 |
| Serum Healthy vs CRC | L-Phenylalanine | 165.0783 | 4.06 | 5.399E-04 | 0.75 | 1.92 | PCCB, COMT, BCAT1, BCAT2 |
| Serum Healthy vs CRC | L-Tryptophan | 187.0633 | 6.2 | 1.416E-11 | 0.51 | 1.66 |  |
| Serum Healthy vs CRC | Hexadecasphinganine | 273.2666 | 21.59 | 2.511E-02 | 1.24 | 1.06 | MTR, MTHFR, TAT, MAT2A |
| Serum Healthy vs CRC | LPC 18:1 | 521.3474 | 33.89 | 4.337E-06 | 2.39 | 1.09 | CHAT, PLD2, PLD1, CHDH |
| Serum Healthy vs CRC | Choline | 103.0998 | 1.45 | 5.746E-07 | 0.52 | 1.53 |  |
| Serum Healthy vs CRC | Pyroglutamic acid | 129.0426 | 2.25 | 3.517E-04 | 0.64 | 1.28 | P4HA2, PYCR1, P4HA1, PYCR2 |
| Serum Healthy vs CRC | Carnitine | 161.1052 | 1.52 | 5.275E-03 | 1.28 | 1.27 |  |
| Serum Healthy vs CRC | LysoPC(O-18:0/0:0) | 509.384 | 40.52 | 1.188E-05 | 0.59 | 1.37 |  |
| Serum Healthy vs CRC | LysoPE(18:0/0:0) | 481.3169 | 38.57 | 1.253E-02 | 1.57 | 1.53 |  |
| Serum Healthy vs CRC | LysoPE(16:0/0:0) | 453.2859 | 33.16 | 2.203E-05 | 1.87 | 1.78 |  |
| Serum Healthy vs CRC | LysoPE(18:2(9Z,12Z)/0:0) | 477.2864 | 31.41 | 1.656E-06 | 1.90 | 1.10 |  |
| Serum Healthy vs CRC | Methionine | 149.0503 | 2.12 | 4.634E-02 | 1.47 | 1.32 |  |
| Serum Healthy vs CRC | LysoPC(16:0/0:0) | 517.2838 | 34.97 | 2.604E-02 | 0.82 | 1.85 |  |
| Serum Healthy vs CRC | Hypoxanthine | 159.1238 | 1.65 | 1.363E-20 | 0.22 | 2.73 | SAT2, SAT1, PAOX |
| Serum Healthy vs CRC | Biomarker A | 113.0585 | 1.59 | 0.045973 | 1.26 | 1.11 |  |
| Serum Healthy vs CRC | Glycocholic acid | 465.3206 | 35.37 | 3.957E-04 | 0.70 | 1.24 |  |
| Serum UC vs CRC | L-Phenylalanine | 165.0783 | 4.06 | 8.243E-10 | 0.59 | 1.88 | TH,FARSA,TAT |
| Serum UC vs CRC | PE(22:2(13Z,16Z)/15:0) | 757.5629 | 48.41 | 2.955E-03 | 1.87 | 1.37 | PLA2G5,PLA2G2F,PLA2G4A |
| Serum UC vs CRC | L-Tryptophan | 187.0633 | 6.2 | 2.560E-07 | 0.58 | 1.92 | DDC,IDO1,TPH1 |
| Serum UC vs CRC | Sphinganine | 301.298 | 24.93 | 2.500E-03 | 1.42 | 1.25 | PPAP2C,PPAP2A,PPAP2B |
| Serum UC vs CRC | Phytosphingosine | 317.2927 | 21.85 | 1.322E-02 | 1.41 | 1.37 | GBGT1,PIGL,PIGQ |
| Serum UC vs CRC | Choline | 103.0998 | 1.45 | 1.948E-07 | 0.58 | 1.91 | CHAT,PLD2,PLD1 |
| Serum UC vs CRC | Pyroglutamic acid | 129.0426 | 2.25 | 9.956E-03 | 0.75 | 1.54 | QPCT,OPLAH,ANG |
| Serum UC vs CRC | Carnitine | 161.1052 | 1.52 | 2.520E-08 | 1.78 | 1.32 | CRAT,XDH,MPO |
| Serum UC vs CRC | Galactaric acid | 227.1881 | 23.59 | 8.247E-03 | 1.45 | 1.10 | UGT2B4,UGT1A4,UGT2B7 |
| Serum UC vs CRC | LysoPE(18:2(9Z,12Z)/0:0) | 477.2864 | 31.41 | 1.197E-02 | 1.38 | 1.22 | ENPP2 |
| Serum UC vs CRC | Linolenic acid | 278.2239 | 45.06 | 2.427E-09 | 2.20 | 1.97 | PLA2G5,PLA2G2F,PLA2G4A |
| Serum UC vs CRC | PC(18:2(9Z,12Z)/18:2(9Z,12Z)) | 781.5743 | 48.41 | 5.573E-03 | 1.55 | 1.11 | LYPLA1,PLA2G15,PLA2G5 |
| Serum UC vs CRC | Hypoxanthine | 159.1238 | 1.65 | 1.239E-17 | 0.26 | 2.98 | XDH，HPRT1，SLC29A2 |
| Serum UC vs CRC | Biomarker A | 113.0585 | 1.59 | 3.434E-03 | 1.49 | 1.54 |  |
| Feces Healthy vs CRC | L-Isoleucine | 131.0924 | 2.59 | 5.116E-06 | 2.41 | 2.39 | BCAT1,BCAT2,IARS |
| Feces Healthy vs CRC | L-Tryptophan | 187.0639 | 6.14 | 1.799E-06 | 0.55 | 2.41 | DDC,IDO1,TPH1 |
| Feces Healthy vs CRC | L-Valine | 117.0759 | 1.72 | 2.259E-05 | 2.18 | 2.36 | PCCB,COMT,BCAT1 |
| Feces Healthy vs CRC | Monoethylhexyl phthalic acid | 278.1534 | 39.52 | 1.232E-02 | 0.76 | 1.64 |  |
| Feces Healthy vs CRC | Methionine | 149.0489 | 2.26 | 7.075E-04 | 2.35 | 1.87 | MTR,MTHFR,TAT |
| Feces Healthy vs CRC | Choline | 103.0998 | 1.45 | 1.412E-04 | 2.14 | 2.20 | CHAT,PLD2,PLD1 |
| Feces Healthy vs CRC | D-Lysine | 146.1051 | 1.39 | 8.017E-03 | 1.97 | 1.68 |  |
| Feces Healthy vs CRC | L-Proline | 98.0343 | 1.65 | 1.236E-04 | 2.07 | 2.29 | P4HA2,PYCR1,P4HA1 |
| Feces Healthy vs CRC | Butylate | 199.0373 | 6.07 | 3.606E-02 | 0.75 | 1.61 |  |
| Feces Healthy vs CRC | 3-Methyl sulfolene | 132.0231 | 2.26 | 4.130E-04 | 1.64 | 2.06 |  |
| Feces Healthy vs CRC | O-Ureidohomoserine | 173.069 | 1.72 | 2.190E-02 | 0.44 | 0.90 |  |
| Feces Healthy vs CRC | Biomarker A | 113.0585 | 1.59 | 2.857E-03 | 2.72 | 1.74 |  |
| Feces Healthy vs CRC | Isonicotinic acid | 101.0491 | 2.26 | 1.085E-02 | 0.71 | 0.99 |  |
| Feces Healthy vs CRC | Tyrosine | 164.0438 | 2.39 | 2.075E-03 | 1.83 | 1.92 |  |
| Feces Healthy vs CRC | 2,5-Dihydro-2,4,5-trimethylthiazole | 152.0341 | 2.26 | 1.742E-02 | 1.48 | 1.57 |  |
| Feces Healthy vs CRC | Hypoxanthine | 159.1238 | 1.65 | 3.07E-02 | 1.79 | 1.01 | XDH,HPRT1,SLC29A2 |
| Feces Healthy vs CRC | N1-Acetylspermidine | 170.1354 | 1.39 | 1.435E-02 | 0.72 | 1.06 | SAT2,SAT1,PAOX |
| Feces Healthy vs CRC | 3-Methylthiophene | 218.0189 | 1.59 | 8.895E-03 | 0.58 | 1.54 |  |
| Feces Healthy vs CRC | 5-Aminopentanoic acid | 100.0512 | 1.72 | 6.321E-04 | 2.03 | 2.33 |  |
| Feces Healthy vs CRC | Fortimicin A | 370.2193 | 8.61 | 1.855E-02 | 0.76 | 1.66 |  |
| Feces UC vs CRC | Urobilin | 594.3333 | 15.44 | 1.780E-10 | 5.07 | 1.68 |  |
| Feces UC vs CRC | Dehydrochenodeoxycholate | 390.2759 | 28.41 | 1.184E-10 | 9.65 | 1.72 | FABP6 |
| Feces UC vs CRC | L-Isoleucine | 131.0942 | 3.06 | 3.486E-11 | 1.42 | 1.06 | BCAT1,BCAT2,IARS |
| Feces UC vs CRC | 3b-Hydroxy-5-cholenoic acid | 374.2806 | 31.02 | 8.767E-09 | 4.34 | 1.73 | FABP6 |
| Feces UC vs CRC | L-Valine | 117.0786 | 1.72 | 1.299E-10 | 1.95 | 1.07 | PCCB,COMT,BCAT1 |
| Feces UC vs CRC | Oleamide | 281.2711 | 47.96 | 2.932E-09 | 0.11 | 2.37 | PLA2G2A,MAOA,DDC |
| Feces UC vs CRC | Isoleucyl-Valine | 230.1618 | 5.93 | 8.117E-03 | 2.3 | 1.33 |  |
| Feces UC vs CRC | 4-Hydroxyproline | 153.039 | 2.45 | 4.929E-02 | 0.52 | 1.87 | DAO,P4HA2,PYCR1 |
| Feces UC vs CRC | LysoPC(16:0/0:0) | 330.2779 | 48.08 | 8.469E-44 | 15.16 | 2.46 | LYPLA1,PLA2G15,PLA2G5 |
| Feces UC vs CRC | LysoPE(0:0/18:3(6Z,9Z,12Z)) | 475.3007 | 10.55 | 1.548E-02 | 0.41 | 1.01 | ENPP2 |
| Feces UC vs CRC | Pipecolic acid | 85.0895 | 3.06 | 7.586E-09 | 0.21 | 1.08 | PIPOX |
| Feces UC vs CRC | LysoPC(18:2(9Z,12Z)/0:0) | 519.3244 | 11.15 | 4.240E-02 | 0.26 | 1.01 | LYPLA1,PLA2G15,PLA2G5 |
| Feces UC vs CRC | Palmitoylglycine | 283.2856 | 53.57 | 8.480E-24 | 3.43 | 1.14 |  |
| Feces UC vs CRC | Monoethylhexyl phthalic acid | 278.1541 | 39.98 | 7.606E-09 | 1.42 | 1.03 |  |
| Feces UC vs CRC | Methionine | 149.0489 | 2.26 | 2.549E-11 | 4.23 | 1.05 | MTR,MTHFR,TAT |
| Feces UC vs CRC | Galactaric acid | 227.1881 | 24.26 | 1.337E-08 | 0.11 | 1.00 | UGT2B4,UGT1A4,UGT2B7 |
| Feces UC vs CRC | Hypoxanthine | 159.1238 | 1.65 | 8.573E-08 | 1.24 | 1.64 | XDH,HPRT1,SLC29A2 |
| Feces UC vs CRC | Biomarker A | 113.0585 | 1.59 | 5.195E-13 | 1.72 | 1.25 |  |

**Table S3**

Serotype identification of *S. flexneri C.11.*

| No. | Polyvalent serum | Type specific serum | | | | | Group factor serum | | |
| --- | --- | --- | --- | --- | --- | --- | --- | --- | --- |
|  |  | 1 | 2 | 3 | 4 | 5 | 3，4 | 6 | 7 |
| 1a | + | + | - | - | - | - | + | - | - |
| 1b | + | + | - | - | - | - | + | + | - |
| 2a | + | - | + | - | - | - | + | - | - |
| 2b | **+** | **-** | **+** | **-** | **-** | **-** | **-** | **-** | **+** |
| 3a | + | - | - | + | - | - | (+) | + | + |
| 3b | + | - | - | + | - | - | + | + | - |
| 4a | + | - | - | - | + | - | + | - | - |
| 4b | + | - | - | - | + | - | - | + | - |
| 5a | + | - | - | - | - | + | + | - | - |
| 5b | + | - | - | - | - | + |  | - | + |
| 6 | + | - | - | - | - | - | (+) | - | - |
| X | + | - | - | - | - | - | - | - | + |
| Y | + | - | - | - | - | - | + | - | - |

**Table S4**

Retention times and MS data of affinity compounds from *S. flexneri C.11* by HPLC-QTOF-MS.

| Peak NO. | RT (min) | m/z | Formula | Identification |
| --- | --- | --- | --- | --- |
| 1 | 6.86 | 150.06 | C_5_H_11_NO_2_S | L-methionine |
| 2 | 36.13 | 245.13 | C_14_H_16_N_2_O_2_ | Cyclo(Phe-Pro) |
| 3 | 29.82 | 197.13 | C_10_H_16_N_2_O2 | Cyclo(Val-Pro) |
| 4 | 8.14 | 235.12 | C_11_H_16_N_4_O_3_ | His-Pro |
| 5 | 17.15 | 166.09 | C_9_H_11_NO_2_ | Phenylalanine |
| 6 | 8.88 | 132.1 | C_6_H_13_NO_2_ | L-Isoleucine |
| 7 | 23.24 | 229.12 | C_10_H_16_N_2_O_4_ | PyroGlu-Val |
| 8 | 16.52 | 211.14 | C_11_H_18_N_2_O_2_ | Cyclo(Pro-Leu) |
| 9 | 19.28 | 227.1 | C_10_H_14_N_2_O_4_ | PyroGlu-Pro |
| 10 | 5.66 | 118.09 | C_5_H_11_NO_2_ | Valine |
| 11 | 0.75 | 104.11 | C_5_H_14_ON^+^ | Choline |
